# Translation Initiation from Conserved Non-AUG Codons Provides Additional Layers of Regulation and Coding Capacity

**DOI:** 10.1128/mBio.00844-17

**Published:** 2017-06-27

**Authors:** Ivaylo P. Ivanov, Jiajie Wei, Stephen Z. Caster, Kristina M. Smith, Audrey M. Michel, Ying Zhang, Andrew E. Firth, Michael Freitag, Jay C. Dunlap, Deborah Bell-Pedersen, John F. Atkins, Matthew S. Sachs

**Affiliations:** a School of Biochemistry and Cell Biology, University College Cork, Cork, Ireland; b Department of Biology, Texas A&M University, College Station, Texas, USA; c Division of Virology, Department of Pathology, University of Cambridge, Cambridge, United Kingdom; d Department of Biochemistry and Biophysics, Center for Genome Research and Biocomputing, Oregon State University, Corvallis, Oregon, USA; e Department of Molecular and Systems Biology, Geisel School of Medicine, Dartmouth College, Hanover, New Hampshire, USA; f Department of Human Genetics, University of Utah, Salt Lake City, Utah, USA; University of California, Berkeley

**Keywords:** *Neurospora*, filamentous fungi, gene regulation, molecular genetics, translational control

## Abstract

*Neurospora crassa cpc-1* and *Saccharomyces cerevisiae GCN4* are homologs specifying transcription activators that drive the transcriptional response to amino acid limitation. The *cpc-1* mRNA contains two upstream open reading frames (uORFs) in its >700-nucleotide (nt) 5′ leader, and its expression is controlled at the level of translation in response to amino acid starvation. We used *N. crassa* cell extracts and obtained data indicating that *cpc-1* uORF1 and uORF2 are functionally analogous to *GCN4* uORF1 and uORF4, respectively, in controlling translation. We also found that the 5′ region upstream of the main coding sequence of the *cpc-1* mRNA extends for more than 700 nucleotides without any in-frame stop codon. For 100 *cpc-1* homologs from Pezizomycotina and from selected Basidiomycota, 5′ conserved extensions of the CPC1 reading frame are also observed. Multiple non-AUG near-cognate codons (NCCs) in the CPC1 reading frame upstream of uORF2, some deeply conserved, could potentially initiate translation. At least four NCCs initiated translation *in vitro*. *In vivo* data were consistent with initiation at NCCs to produce N-terminally extended *N. crassa* CPC1 isoforms. The pivotal role played by CPC1, combined with its translational regulation by uORFs and NCC utilization, underscores the emerging significance of noncanonical initiation events in controlling gene expression.

## IMPORTANCE

There is a deepening and widening appreciation of the diverse roles of translation in controlling gene expression. A central fungal transcription factor, the best-studied example of which is *Saccharomyces cerevisiae* GCN4, is crucial for the response to amino acid limitation. Two upstream open reading frames (uORFs) in the GCN4 mRNA are critical for controlling GCN4 synthesis. We observed that two uORFs in the corresponding *Neurospora crassa cpc-1* mRNA appear functionally analogous to the GCN4 uORFs. We also discovered that, surprisingly, unlike GCN4, the CPC1 coding sequence extends far upstream from the presumed AUG start codon with no other in-frame AUG codons. Similar extensions were seen in homologs from many filamentous fungi. We observed that multiple non-AUG near-cognate codons (NCCs) in this extended reading frame, some conserved, initiated translation to produce longer forms of CPC1, underscoring the significance of noncanonical initiation in controlling gene expression.

## INTRODUCTION

General amino acid control (GAAC) in fungi activates amino acid biosynthetic gene expression in response to amino acid limitation (1, 2). This regulatory pathway was originally called cross-pathway control in *Neurospora crassa* and general control in *Saccharomyces cerevisiae* (3). *N. crassa cpc-1* and *S. cerevisiae GCN4* specify homologous bZIP transcription factors that were identified using forward genetics based on their function to transcriptionally activate amino acid biosynthetic genes in response to amino acid limitation or imbalance.

Both *N. crassa* CPC1 and yeast Gcn4p contain a transcription activation domain, a basic DNA binding domain, and a leucine zipper region involved in dimerization. Genes regulated by CPC1 or GCN4 contain the general control response element (GCRE) sequence TGA(C/G)TCA or a similar sequence (3, 4). A comparative study of *S. cerevisiae* Gcn4p, *Candida albicans* Gcn4p, and *N. crassa* CPC1 revealed that many genes were regulated by these factors in each organism and that the common core of regulated genes was mostly amino acid biosynthetic genes (5). *N. crassa cpc-1*, like *Aspergillus nidulans cpcA* and *C. albicans GCN4* but unlike *S. cerevisiae GCN4*, appears transcriptionally autoregulated in response to amino acid limitation (5–8), and these fungal *cpc-1* genes contain GCRE sequences in their 5′ regions implicated in transcriptional autoregulation.

The translational control of *GCN4* in response to amino acid limitation is the canonical example of how upstream open reading frames (uORFs) mediate regulation of translation via control of reinitiation (1, 9, 10). Four uORFs affect the progression of ribosomes through the 5′ leader of *GCN4* mRNA to regulate *GCN4* expression in response to amino acid limitation. uORF1 acts as a positive regulatory element to facilitate reinitiation, while uORF4 strongly inhibits the translation of *GCN4*. uORF2 and uORF3 play relatively minor roles. *In vivo* experiments (11) and cell-free translation assays (12) confirm that translation of uORF1 generates reinitiating ribosomes that can start translation at either uORF4 or GCN4 and that translation of uORF4 is incompatible with reinitiation at the GCN4 start codon. The phosphorylation of initiation factor eIF2α (α subunit of eukaryotic initiation factor 2) by the GCN2 kinase in response to amino acid limitation causes ribosomes to scan past uORF4 and to increase reinitiation at the GCN4 start codon. Fungal homologs of *GCN4* contain at least two uORFs, and it is generally thought that these perform similar functions as *GCN4* uORF1 and uORF4. *ATF4*, a mammalian homolog of *GCN4*, also contains two uORFs, and these also function similarly to *GCN4* uORF1 and uORF4 (13, 14).

*N. crassa cpc-1* expression is known to be translationally controlled in response to histidine limitation as determined by polysome association analyses (15). Also, *N. crassa cpc-3*, the functional homolog of *S. cerevisiae GCN2*, is required for the GAAC response, and disruption of *cpc-3* abolishes the increase of CPC1 protein in response to amino acid starvation (16). These studies are consistent with translational regulation of *cpc-1* through its uORFs occurring similarly to that of *S. cerevisiae GCN4*.

An additional consideration for regulation of *cpc-1* is the discovery that the CPC1 reading frame could be extended at its amino terminus if a near-cognate non-AUG start codon (NCC) was used to initiate translation (17). NCCs are known to be used as initiation codons (18–23), and their significance is actively being explored (24–28). In other organisms, the use of NCCs appears to increase in response to conditions that reduce the stringency of start codon selection (29–32).

Here, we used an *N. crassa* cell-free translation system to show that *N. crassa cpc-1* uORF1 and uORF2 act analogously to uORF1 and uORF4, respectively, in *S. cerevisiae GCN4* in that ribosomes reinitiate efficiently after translating uORF1 but not uORF2. We also discovered and identified conserved potential N-terminal extensions in the *cpc-1* homologs from a much larger group of fungi, including Pezizomycotina and Basidiomycota, but not yeast. Multiple NCCs, some well conserved and in optimal initiation contexts, which potentially initiate the extension of the *N. crassa cpc-1* homolog were examined both *in vitro* and *in vivo*. The positions of these NCCs indicate that their utilization could bypass the translational inhibitory effect of uORF2. We observed that four of the identified NCCs were used *in vitro* and that, as predicted, their use abrogated the inhibitory effect of uORF2. Evidence for NCC utilization *in vivo* was also obtained. These findings indicate that, in addition to translational control via uORFs, the filamentous fungi possess other translational mechanisms to produce different CPC1 isoforms.

## RESULTS

### Bioinformatic analyses of fungal *cpc-1.*

**FIG 1:**
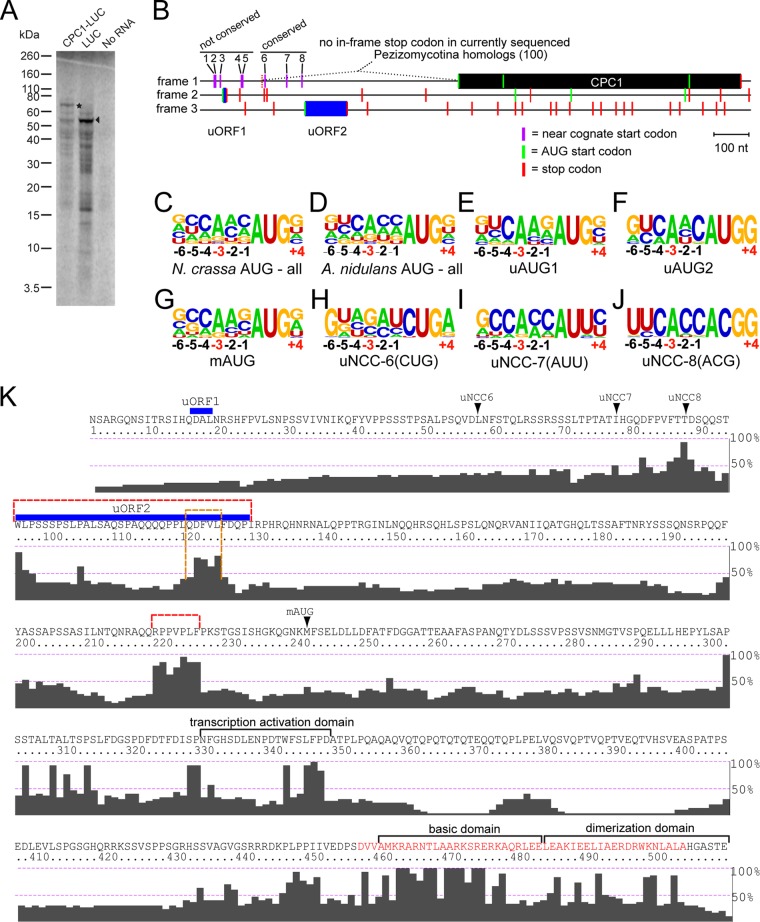
N-terminal extensions of Pezizomycotina CPC1. (A) *cpc-1–luc* mRNA produces a larger product *in vitro* than *luc* mRNA. mRNA templates were used to program *N. crassa in vitro* translation extracts, and [^35^S]Met-labeled products were analyzed on a 12% NuPAGE gel. Translation of mRNA for *N. crassa cpc-1–luc* (in which the 5′ leader of *cpc-1* plus the first three codons of its mORF are fused in frame to firefly luciferase) and that for *luc* were compared to a no-mRNA control. The positions of full-length firefly LUC (arrowhead) and the larger CPC1-LUC product (asterisk) are indicated. (B) Schematic diagram of the *N. crassa cpc-1* mRNA. Each reading frame is on a separate line. Frame 1 specifies CPC1 (black rectangle). uORF1and uORF2 (blue rectangles) initiate from uAUG1 and uAUG2, respectively, in other reading frames. AUG codons are indicated by green bars, and stop codons are indicated by red bars. NCCs in frame 1 upstream of uORF2 are indicated by magenta bars and are numbered 1 to 8. The approximate position of the 3′-most stop codon upstream of uORF2 and in frame with the main ORF (present in *Cordyceps bassiana*) is indicated (dashed red bar). The number of sequences used for comparisons is shown in parentheses. Features are drawn to scale. The nucleotide sequence of the *N. crassa cpc-1* 5′ leader is given in Fig. S6 in the supplemental material. (C to J) Frequency WebLogo of the conservation of the initiation contexts, from −6 to +4, of all *N. crassa* genes initiated with AUG (C), all *A. nidulans* genes initiated with AUG (D), Pezizomycotina *cpc-1* uAUG1 (E), Pezizomycotina *cpc-1* uAUG2 (F), Pezizomycotina *cpc-1* mAUG (G), Pezizomycotina NCC6 (CUG) (H), Pezizomycotina NCC7 (AUU) (I), and Pezizomycotina NCC8 (ACG) (J). Letter heights are proportional to the frequency of occurrence of each nucleotide at each position. The crucial positions −3 and +4 are indicated in red underneath the frequency plots. Data used to calculate consensus AUG initiation context for *N. crassa* and *A. nidulans* were obtained from the Transterm database (61). (K) The amino acid sequence encoded by the *N. crassa* mRNA in the CPC1 reading frame, starting from the 5′ end of the mRNA and ending with the first in-frame stop codon. mAUG indicates the annotated *cpc-1* initiation codon; upstream NCC6 (uNCC6), uNCC7, and uNCC8 are also indicated. The approximate positions of uORF1 and uORF2 (which are in other reading frames) are indicated by blue lines. The C-terminal bZIP domain of CPC1 is indicated in red font. The regions analyzed in Fig. S3A and B are bracketed by dashed red lines. The highly conserved patch specifically marked in Fig. S3A is bracketed by a dashed orange line. The level of conservation of each residue from the alignment of homologs from 95 species (those used to construct the tree in Fig. S1A) is shown below the amino acid sequence and was generated using ClustalX2. The conservation, expressed as percent amino acid identity, is indicated on the right side of each alignment.

While studying the regulation of *cpc-1* by its uORFs in *N. crassa* extracts, using a construct in which the wild-type (WT) 5′ leader of *cpc-1* was fused with the open reading frame of firefly luciferase (LUC), we observed a band of predicted size and a band ~20 kDa larger than predicted (Fig. 1A). This prompted a more careful examination of the mRNA 5′ leader sequence. We found that the CPC1 reading frame extended far upstream (Fig. 1B), without any in-frame stop codons, to the major mapped transcription initiation site, which is located 703 nucleotides (nt) 5′ of the predicted AUG for the main open reading frame (designated mAUG and mORF, respectively). We previously noted that *N. crassa cpc-1* could hypothetically use upstream near-cognate start codons for initiation (17). We next compiled partial or complete sequences of *cpc-1* homologs from 108 Pezizomycotina species: 100 sequences included the region spanning from uORF1 to a position downstream of the mAUG and were analyzed further. All homologs contain two AUG-initiated uORFs, with uORF1 spanning 3 to 6 codons and uORF2 spanning 35 to 70 codons, including their stop codons. Surprisingly, in all cases, the reading frame for CPC1 could be substantially N-terminally extended without encountering an in-frame stop. The shortest extension of the CPC1 ORF without encountering an in-frame stop codon is 160 codons in *Leptosphaeria maculans*. We note that some automated annotations of CPC1 homologs include this N-terminal extension (e.g., XP_001906068, EGR46729, and EKJ70155), but annotations do not resolve where initiation occurs. The presence of this feature in both Sordariomycetes and Eurotiomycetes suggests that it was present in their last common ancestor and possibly earlier; the last common ancestor of all Pezizomycotina is estimated to have lived at least 320 million years ago (33).

Based on the mechanism of translational control of *S. cerevisiae GCN4*, control of *cpc-1* would involve ribosomes initiating at uORF1 and reinitiating at uORF2 under amino acid-sufficient conditions. When eIF2α phosphorylation levels increase in response to amino acid limitation, ribosomes would reinitiate at the downstream *cpc-1* mAUG instead of uORF2. Remarkably, without exception in the Pezizomycotina, there is no stop codon in the reading frame of the mORF between the uORF2 AUG and the mAUG. The in-frame stop codon closest to uAUG2 (*Cordyceps bassiana*) is 101 nt upstream of it. Thus, the potential amino-terminal extensions of CPC1 are encoded upstream of uORF2 (Fig. 1B).

Initiation upstream of the uORF2 AUG could produce N-terminally extended isoforms of CPC1 whose synthesis would not be subject to inhibition by uORF2. We searched for potential start codons in this region of *N. crassa cpc-1* mRNA that were in frame with the predicted mAUG. Eight NCCs fulfilling these criteria were identified—three AUC (NCC1, NCC3, and NCC4), two ACG (NCC2 and NCC8), two AUU (NCC5 and NCC7), and one CUG codon (NCC6) (Fig. 1B). We next searched for potential NCCs in similar regions of *cpc-1* transcripts from all Pezizomycotina (see Fig. S1 in the supplemental material) and compared their conservation levels. Three NCCs in *N. crassa* showed particularly deep conservation—the closest to the CPC1 AUG, an ACG (NCC8), is perfectly conserved in 98 of 100 species; NCC7, an AUU, is conserved in 74 of 100 species (as AUU in 64 and AUC or AUA in 10); NCC6, a CUG, is conserved in 77 of 100 species (as CUG in 73 and UUG in 4) (Fig. S1 and S2). In no case was there an in-frame stop codon between these three conserved NCCs and the mAUG. The three conserved NCCs also show a clear pattern of fungal branch-specific distribution: the minority of homologs lacking both AUU and CUG NCCs clustered separately from the other homologs (Fig. S1). The other five NCCs showed sporadic conservation and were found only in species that were closely related to *N. crassa*. None of the *N. crassa* NCCs appeared conserved in the two most distant Pezizomycotina, *Arthrobotrys oligospora* and *Tuber melanosporum* (Fig. S1). However, even these two species’ CPC1 homologs contain multiple NCCs upstream of uORF2 and in frame with the mORF—6 NCCs in *A. oligospora* and 7 NCCs in *T. melanosporum* (Fig. S2C).

10.1128/mBio.00844-17.1 FIG S1  Superimposing conservation of NCCs in *cpc-1* homologs on the phylogenetic trees of Pezizomycotina and Agaricomycetes. (A) Phylogeny of Pezizomycotina based on CPC1 amino acid sequences and conservation of NCC6, NCC7, and NCC8. The unrooted phylogenetic tree is based on the amino acid sequences (from the 5′ end to the mORF stop codon when no in-frame stop codon is present or from the stop codon in the 5′ region to the mORF stop codon) of the *cpc-1* homologs from the Pezizomycotina species indicated on the right of each branch. Species names are coded with respect to which NCCs are identical to *N. crassa*: black, NCC6, NCC7, and NCC8; blue, NCC7 and NCC8; green, NCC6 and NCC8; red, NCC8; orange, no identical NCCs. (B) Phylogeny of Agaricomycetes based on CPC1 amino acid sequences and conservation of a single NCC. The unrooted phylogenetic tree is based on the amino acid sequences (from the 5′ end to the mORF stop codon or from the stop codon in the 5′ region to the mORF stop codon) of the *cpc-1* homologs from the Agaricomycetes species indicated on the right of each branch. The asterisk indicates a sequence presumed to derive from an unknown Agaricomycetes species contaminating the *Pinus taeda* EST library. Species names in black, NCC = AUU; names in blue, NCC = UUG; in orange, NCC = AUA; in red, NCC = ACG or CUG. The trees were generated using ClustalX. Bootstrap values are given for key nodes in Pezizomycotina. The scale represents the divergence rate at each residue. Download FIG S1, PDF file, 0.2 MB. Copyright © 2017 Ivanov et al. 2017 Ivanov et al. This content is distributed under the terms of the Creative Commons Attribution 4.0 International license.

10.1128/mBio.00844-17.2 FIG S2  Conservation of NCCs in *cpc-1* homologs of different fungi. (A and B) Nucleotide sequences used for generating WebLogo in Fig. 1 and S5, respectively. Species identifiers of the sequences used are indicated on the left. An asterisk next to the species name indicates sequence presumed to derive from unknown Pezizomycotina or Agaricomycetes that is contaminating an EST library of the indicated species (e.g., Agaricomycetes sequence contaminating *Pinus taeda* library). Presumed start codons are highlighted in green. Sequences from species names in quotation marks are presumed contaminants of fungal origin because they cluster with *cpc-1* from Pezizomycotina or Agaricomycetes even though the name of the sequenced species is a plant. Alignments of the 10 nucleotides including the AUG or NCC start codons and the nucleotides that define the initiation contexts (−6 to +4) which were used to generate the WebLogo in Fig. 1C to J and S5D and E are shown for *cpc-1/GCN4* homologs from uAUG1 uAUG2, mAUG, NCC6, NCC7, and NCC8 from Pezizomycotina (A) and the single NCC from Agaricomycetes (B). (C) Nucleotide sequences of the 5′ regions of *cpc-1* homologs from the upstream in-frame stop codon to the uAUG in *Tuber melanosporum* and *Arthrobotrys oligospora*. NCCs are highlighted in green. Download FIG S2, PDF file, 0.3 MB. Copyright © 2017 Ivanov et al. 2017 Ivanov et al. This content is distributed under the terms of the Creative Commons Attribution 4.0 International license.

We next examined the conservation of the initiation contexts for the three conserved NCCs and for the uORF1 AUG (uAUG1), uORF2 AUG (uAUG2), and mAUG. The preferred initiation context in *N. crassa* (Fig. 1C), which is considered optimal, is similar to the preferred context in the relatively distant *Aspergillus fumigatus* (34) and *Aspergillus nidulans* (as shown in Fig. 1D). uAUG1 and uAUG2 are in conserved optimal contexts (Fig. 1E and F and S2A), consistent with their presumed roles in regulating CPC1 translation through controlling reinitiation. Conservation of mAUG context is weaker, but the consensus is still near optimal (Fig. 1G). Of the three conserved NCCs, NCC8, which is closest to the mAUG and is the most conserved, showed the highest context conservation (Fig. 1J and S2A). The consensus initiation context of NCC8 in species that we examined is nearly optimal (nucleotides −4, −3, −1, and +4 match the consensus). The most important nucleotides, A at position −3 and G at position +4, are perfectly conserved in all Pezizomycotina that have NCC8. Lower context conservation is observed for NCC7 and NCC6 (Fig. 1H and I), although their consensus initiation contexts remain nearly optimal.

One question raised by the potential N-terminal extensions of *cpc-1* homologs is whether they are evolutionarily conserved at the amino acid sequence level. A plot of the amino acid conservation of Pezizomycotina CPC1 sequences relative to *N. crassa* sequence is shown in Fig. 1K. A highly conserved region is present near the C terminus of CPC1 (residues 430 to 500), which corresponds to the α-helix of the bZIP DNA binding domain (Fig. 1K). Excluding this, there are few highly conserved stretches, but the N-terminal extensions are as well conserved as the mORF. We examined two conserved regions in the N-terminal extension (Fig. 1K, red dashes) more closely to determine if the conservation is at the amino acid or the nucleotide level and, if it is at the amino acid level, which reading frame showed the highest conservation (Fig. S3A and B). The first conserved region examined overlaps uORF2. The proportion of synonymous substitutions was much higher in the mORF frame than in uORF2 frame (Fig. S3A). For the 5 codons showing the highest amino acid conservation in the mORF frame (Fig. 1K, orange dashed bracket), the ratio of synonymous to nonsynonymous substitutions is particularly striking. The second conserved region examined comprises 7 codons starting 16 codons upstream of the mAUG. This region also shows a high proportion of synonymous substitutions in the mORF frame compared to the other frames (Fig. S3B), indicating that its conservation occurs because of selection in the mORF frame.

10.1128/mBio.00844-17.3 FIG S3  Regions upstream of mAUG show coding conservation in frame with the mORF. (A) Conservation in the N-terminal extension of *N. crassa cpc-1*. (i and ii) Nucleotide and codon alignments of the region corresponding to uORF2 of *cpc-1* in *N. crassa* are shown in frame with the main ORF (frame 1 in Fig. 1B) (i) and in frame with uORF2 (frame 3 in Fig. 1B) (ii). (iii to v) A proline-rich region (residues 218 to 224, red dashed bracket in Fig. 1K) upstream of the *cpc-1* mAUG shows high conservation in the mORF. Nucleotide and codon alignments of this region are shown for frame 1 (in frame with the mORF) (iii), frame 2 (iv), and frame 3 (v). In these alignments, consensus codons (and gaps) are highlighted in white, synonymous codons in green, nonsynonymous codons in brown, and stop codons in magenta. Species identifiers of the sequences used are indicated on the left. The numbers of synonymous and nonsynonymous substitutions are summed below each panel. The additional numbers in parentheses below panel i indicate the number of synonymous and nonsynonymous substitutions in frame with the mORF in the small patch of sequence marked by a dashed orange bracket above the aligned sequences. This patch represents the conserved peptide region indicated by the corresponding dashed orange bracket in Fig. 1K. The alignments were generated by a custom script that conceptually translated the main ORF frame into peptide sequence, then performed ClustalX alignment of peptide sequence, and then converted this alignment back to nucleotides. The most common codon in each column was then designated the consensus codon; synonymous and nonsynonymous substitutions were determined relative to it. (C) The region corresponding to the beginning of uORF3 of *Coprinus cinerea cpc-1* is conserved at the amino acid level in frame with the mORF in Agaricomycetes homologs. Nucleotide and codon alignments of the region, including the beginning of uORF3 of *C. cinerea cpc-1* (indicated by red dashed lines in Fig. S5F), are shown for frame 1 (in frame with the main ORF) (vi), frame 2 (in frame with uORF3) (vii), and frame 3 (viii). Species identifiers of the sequences used are indicated on the left. “Pinus_taeda_cont” indicates a sequence coming from an unknown Agaricomycetes species contaminating the *Pinus taeda* EST library. The numbers of synonymous and nonsynonymous substitutions are indicated on the right of each panel. The position of the AUG start codon of uORF3 is indicated by an inverted red triangle. Coloring scheme, alignment generation, and display are the same as in other panels. Download FIG S3, PDF file, 2.6 MB. Copyright © 2017 Ivanov et al. 2017 Ivanov et al. This content is distributed under the terms of the Creative Commons Attribution 4.0 International license.

The coding potential of the upstream extension was analyzed with MLOGD (35). MLOGD calculates coding potential by using the patterns of substitutions observed across a sequence alignment to compare a coding model with a noncoding model via a likelihood-ratio test. When applied in a 20-codon sliding window, MLOGD detected a positive coding signature within the CPC1 AUG-initiated ORF (as expected) and upstream throughout the extension as far 5′ as NCC6 (Fig. S4). The coding signature was weaker (but still positive) from NCC6 to around one-third of the way through uORF2. This may be a result of increased CPC1 frame synonymous site conservation in this region (Fig. S4), leading to fewer sequence variations for MLOGD to distinguish between the coding and noncoding models. The enhanced synonymous site conservation (Fig. S4) is indicative of overlapping functional elements putting extra constraints on sequence evolution in this region, likely including the initiation contexts of NCC6 to NCC8 and the overlapping uORF2. The ratio of nonsynonymous to synonymous substitutions, *dN/dS*, was calculated for the region between NCC8 and the CPC1 AUG using codonml (36) and found to be 0.348 + 0.033 and thus statistically significantly less than 1 (99% confidence interval, 0.26 to 0.43), indicating that the upstream extension is indeed subject to purifying selection at the amino acid level, consistent with its being a coding sequence. Since synonymous site conservation interferes with use of *dS* as a proxy for neutral evolution, we also calculated *dN/dS* for the region from 21 codons after NCC8 to the CPC1 AUG (Fig. S4), giving a *dN/dS* ratio of 0.285. For comparison, the *dN/dS* ratio for the region between the CPC1 AUG and CPC1 stop codon was found to be 0.144 + 0.012, indicating stronger purifying selection on average in the AUG-initiated CPC1 ORF than in the upstream extension. Taken together, these data indicate that the mRNA sequences specifying the N-terminal extension are under purifying selection in the mORF frame.

10.1128/mBio.00844-17.4 FIG S4  Coding potential and synonymous site conservation in *cpc-1*. At the top is a map of the *N. crassa* maximal CPC1-frame ORF region, showing the main CPC1 ORF; uNCC6, uNCC7, and uNCC8; and uORFs 1 and 2. Immediately below is shown the coding potential in the three reading frames as measured with MLOGD using an alignment of the maximal CPC1-frame ORF regions from 96 fungal species and a 20-codon sliding window. MLOGD uses a principle similar to the *dN/dS* statistic (ratio of nonsynonymous to synonymous substitutions) but also accounts for conservative amino acid substitutions being more probable than nonconservative substitutions. Positive scores indicate that the sequence is likely to be coding in the given reading frame and are observed throughout CPC1 and most of the upstream extension, at least from uNCC8. Positions where >50% of the alignment divergence is missing due to alignment gaps are omitted. Below this is shown alignment divergence (aln. divg, mean number of substitutions per nucleotide) for the sequences that contribute to the statistics at each position in the alignment (in any particular column, some sequences may be omitted from the statistical calculations due to alignment gaps). The bottom panels show the analysis of synonymous site conservation across the alignment. The brown line (“o/e”) indicates the ratio of the observed number of synonymous substitutions within a 5-codon window to the number expected under a null model of neutral evolution at synonymous sites, while the red line (“p-val”) depicts the corresponding statistical significance. Download FIG S4, PDF file, 0.1 MB. Copyright © 2017 Ivanov et al. 2017 Ivanov et al. This content is distributed under the terms of the Creative Commons Attribution 4.0 International license.

We next investigated the architecture of *cpc-1* homologs in fungi outside the Pezizomycotina. Examination of multiple sequences from other classes within the Ascomycota, including Saccharomycotina (including *S. cerevisiae GCN4*) and Taphrinomycotina, showed that these *cpc-1* homologs lack the analogous N-terminal extensions of the main ORF. Thus, the conserved N-terminal extension in Ascomycota is confined to Pezizomycotina. Little comparative sequence information was available to examine other fungal phyla except for Basidiomycota. Within this phylum, analysis was complicated by the presence of multiple *cpc-1* paralogs in some species. Typically, the 5′ leaders of *cpc-1* homologs from Basidiomycota have 3 to 4 uAUGs. These can either initiate, or exist within, the reading frames of two or three uORFs (Fig. S5). uORF1 is 4 to 7 codons long, while one of the downstream uORFs, initiated by AUG in a good context, is much longer (uORFL). Crucially, uORFL sometimes overlaps the mORF (see Fig. S5B and C). Examination of 32 *cpc-1* Basidiomycota mRNA sequences (3 from Ustilaginomycotina, 27 from Agaricomycetes, and 2 from Microbotryomycetes) with identifiable uORFs revealed that, in all cases, no stop codon in frame with the mORF is present between uORF1 and the mAUG (Fig. S5A and B). In fact, no stop codon in frame with the mORF is closer than 77 nucleotides upstream of uORF1. Unlike in Pezizomycotina, where three highly conserved NCCs were identified for most N-terminal extensions, no well-conserved NCCs were identified in Basidiomycota. However, in every Basidiomycota *cpc-1* homolog, several NCCs in good initiation contexts and in the same frame with the mORF are present 5′ of the apparently inhibitory uORFL. In all cases, the first NCC is located 5′ of uORF1 such that potential translation initiation at the NCC would bypass the regulatory effects of the uORFs.

We searched the 27 uORF-containing *cpc-1* homologs from Agaricomycetes for conserved features. In these, there is a single conserved NCC capable of initiating translation of an N-terminal extension and this NCC is present at least 31 nucleotides 5′ of the uORF1 AUG (Fig. S5B). Although the position of this NCC is well conserved, its identity is not. In most cases, it is AUU; in others, it is UUG, AUA, or CUG (Fig. S1B). The specific identities of these NCCs appear largely specific to phylogenetic branches.

10.1128/mBio.00844-17.5 FIG S5  Features of *cpc-1* homologs from Basidiomycota. (A to C) Diagrams of the *cpc-1* transcripts from different Basidiomycota branches. AUG codons (green bars), stop codons (red bars), mORFs (black bars), and uORFs (blue bars) are indicated. The numbers of sequences used for the comparisons are shown in parentheses. Features are drawn to scale. (A) *Ustilago maydis* (representing the three available Ustilaginomycotina sequences). Conserved NCCs in frame 1 upstream of uORF2 are indicated by magenta bars. (B) *Coprinopsis cinerea* (representing the 27 available Agaricomycetes sequences containing uORFs). The position of the single conserved NCC in frame 1 upstream of uORF3 is indicated by a magenta bar. (C) *Leucosporidium scottii* (representing the two available Microbotryomycetes sequences containing uORFs). The position of the single conserved NCC in frame 1 upstream of the uORF is indicated by a magenta bar. (D and E) Frequency WebLogo of the conservation of the initiation contexts, from −6 to +4, of all *C. cinerea* genes (D) and the single conserved NCC in frame 1 of Agaricomycetes upstream of the uORFs (E). (F) The amino acid sequence encoded by frame 1 of *C. cinerea cpc-1* mRNA (the mORF), bounded by two in-frame stop codons. mAUG indicates the annotated initiation codon; the position of the conserved upstream NCC is also indicated. The approximate positions of uORF1, uORF2, and uORF3 are indicated by blue lines. The C-terminal bZIP domain is indicated in red font. The region analyzed in Fig. S3C is bracketed by a dashed red line. The conservation of each residue from the alignment of homologs from 27 species (those used to construct the tree in Fig. S1B) is shown below the amino acid sequence. Download FIG S5, PDF file, 0.3 MB. Copyright © 2017 Ivanov et al. 2017 Ivanov et al. This content is distributed under the terms of the Creative Commons Attribution 4.0 International license.

The preferred initiation context in Agaricomycetes, as determined by analyses of *Coprinopsis cinerea* (Fig. S5D), is similar to the context in both Pezizomycotina and mammals. Based on this, the context of the single conserved NCC in the 5′ leaders of *cpc-1* homologs in Agaricomycetes appears favorable if not optimal (Fig. S5E). The putative N-terminal extensions in Agaricomycetes are shorter than in Pezizomycotina—approximately 120 versus 180 amino acids, respectively. The amino acid conservation in Agaricomycetes is also concentrated in the C-terminal region of the mORF that contains the α-helix including the bZIP DNA binding domain (red letters in Fig. S5F). Patches of substantial conservation are observed within the 50 amino acids upstream of the mORF. The most highly conserved stretch in this region was subjected to a more careful examination (Fig. S3C, red dashed line). This sequence overlaps the last, and usually longest, uORF. This analysis indicates that conservation of amino acid sequence of the N-terminal extension in the mORF frame is more important than conservation in the uORF or the third reading frame (Fig. S3C), consistent with the findings in Pezizomycotina.

A peculiar mRNA architecture exists in *cpc-1* homologs in Microbotryomycetes (Fig. S5C). Even though only two uORF-containing homologs of *cpc-1* were obtained in this branch of Basidiomycota, both transcripts have the same unusual feature (Fig. S5C). Unlike Ustilaginomycotina (Fig. S5A) or Agaricomycetes (Fig. S5B), no evidence was detected for the existence of a short regulatory uORF1. The 5′ end of the homolog from *Leucosporidium scottii* is well supported by several expressed sequence tags (ESTs), and the upstream neighboring gene is in close proximity. Thus, instead of a uORF1, a long uORF initiated by AUG in a favorable initiation context is present, which overlaps the mORF. No in-frame stop codons are seen upstream of the mAUG. A single conserved NCC can be identified upstream of the uORF start codon so that ribosomes initiating from this NCC could synthesize an N-terminally extended CPC1 isoform and completely bypass any inhibitory effects of the uORF.

### Experimental analyses of *N. crassa cpc-1.*

To investigate the effects of uORF1, uORF2, and upstream NCCs on the translation of *N. crassa* CPC1 in cell extracts, the 5′ leader of *cpc-1*, including the first two codons of the mORF, was fused in frame to firefly luciferase (*cpc-1–luc*, designated wild type [WT] [Fig. S6]). The functions of initiation codons identified by bioinformatics approaches were tested by mutational analyses of this construct. A UAA mutation (designated UAA) was introduced in frame with, and 12 nt upstream of, the mAUG to terminate translation and therefore truncate translation products that initiated from upstream NCCs. The start codon of uORF1 was mutated to AAA (ΔuORF1), that of uORF2 was mutated to ACA (ΔuORF2), and that of the mORF was mutated to CTC (ΔmAUG), to eliminate their initiation activity.

10.1128/mBio.00844-17.6 FIG S6  The sequences of *cpc-1–luc* fusion constructs used for *in vitro* experiments. Mutations and selected unique restriction sites are shown below the sequence. uORF1, uORF2, and the beginning of the main ORF (mORF) are shaded gray. NCCs are shaded magenta and are numbered 1 to 8. The position of the introduced TAA mutation that terminates the translation from NCCs is indicated by a red box. Luciferase was placed in frame with CPC1 at the XhoI site. Download FIG S6, PDF file, 0.2 MB. Copyright © 2017 Ivanov et al. 2017 Ivanov et al. This content is distributed under the terms of the Creative Commons Attribution 4.0 International license.

**FIG 2:**
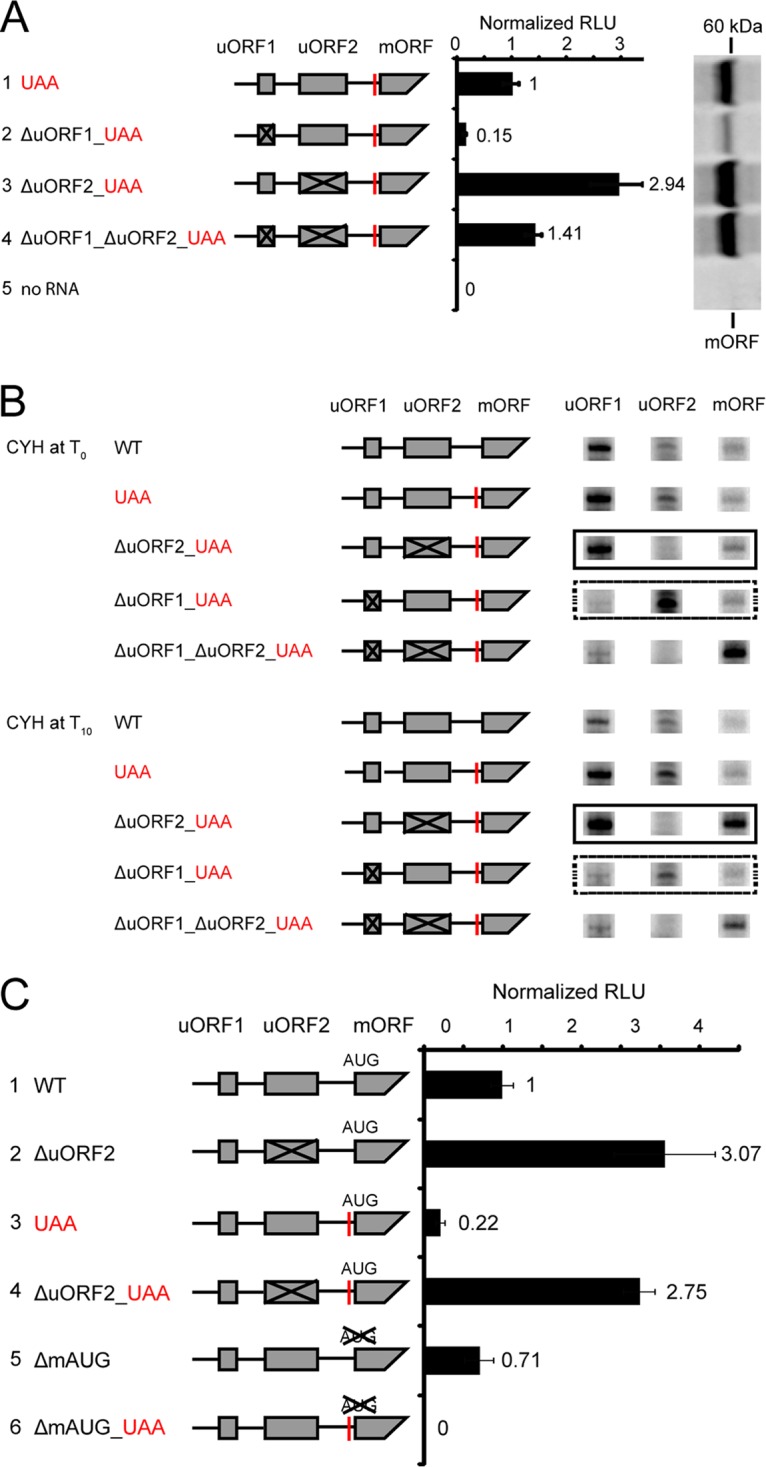
Contribution of *cpc-1* uORF1 and uORF2 to the regulation of translation from the mAUG in *N. crassa* cell extracts. (A) Effects of eliminating *cpc-1* uORF1 and uORF2 on translation from the mAUG. Constructs (numbered 1 to 4) contained the UAA stop codon (red bar) to eliminate translation from upstream in-frame NCCs and the indicated mutations to uORF start codons to eliminate initiation from them (uORF1 AUG to AAA and/or uORF2 AUG to ACA). Capped and polyadenylated mRNA (6 ng) was used to program *N. crassa* translation reaction mixtures (10 µl). LUC activity produced from mRNAs 2 to 4 obtained after 30 min of incubation at 26°C was calculated relative to the activity produced from mRNA 1. Mean values and standard deviations from three independent experiments, each performed in triplicate, are given as normalized relative light units (RLU). In addition, [^35^S]Met-labeled translation products from translation reactions programmed with mRNAs 1 to 4 or with no mRNA were analyzed on 12% NuPAGE gels, and a representative gel is shown. The position of radiolabeled LUC produced from the mAUG is indicated. (B) Toeprint analysis indicates reinitiation following translation of *cpc-1* uORF1 but not uORF2. *cpc-1–luc* mRNA (60 ng) was used to program 20-µl *N. crassa* cell-free translation reaction mixtures. WT mRNA containing the wild-type *cpc-1* 5′ leader and the mRNAs used in panel A were analyzed in parallel along with controls. Reaction mixtures were incubated at 26°C min with cycloheximide (CYH) added either prior to incubation (*T*_0_) or after 10 min of incubation (*T*_10_) as indicated. Radiolabeled primer CPC101 was used to examine ribosomes at uORF1 and uORF2; primer ZW4 was used to examine ribosomes at the mORF. The original data from which the toeprint signals were excised are shown in Fig. S7. (C) Discriminating translation from *N. crassa cpc-1* NCCs and mAUG *in vitro*. Capped and polyadenylated mRNA (6 ng) was used to program *N. crassa* translation reaction mixtures (10 µl) with the indicated constructs. Firefly luciferase activity from each mRNA obtained after 30 min of incubation at 26°C was calculated relative to synthesis from the WT construct. Mean values and standard deviations from three independent experiments, each performed in triplicate, are plotted.

The functions of uORF1 and uORF2 were examined by mutating their start codons separately or together. First, we examined these mutations in constructs containing the UAA mutation to look specifically at luciferase synthesis from the mAUG. Luciferase synthesis was measured by enzyme activity assay and by labeling with [^35^S]Met (Fig. 2A). Compared to a construct containing both uORFs, the ΔuORF1 mutation diminished translation of the mORF as indicated by a reduced level of luciferase activity (15%) and decreased production of [^35^S]Met-labeled polypeptides (compare constructs 1 and 2, Fig. 2A). The ΔuORF2 mutation increased translation from the mAUG approximately 2.9-fold (compare constructs 1 and 3, Fig. 2A). For the ΔuORF1 ΔuORF2 double mutant, the synthesis of luciferase increased (compare constructs 1 and 4, Fig. 2A), but this increase was less than for ΔuORF2 alone. These data suggest that reinitiation occurs after translation of uORF1, that translation of uORF2 is inhibitory, and that a fraction of ribosomes that translate uORF1 reinitiate at uORF2. In the absence of uORF1 and uORF2, synthesis of luciferase is lower than in the absence of uORF2 alone. This could be explained if the NCCs are used more efficiently in the absence of uORF1 (see below).

In earlier studies on *S. cerevisiae GCN4*, we used toeprint analyses to demonstrate reinitiation following uORF1 but not uORF4 translation in *S. cerevisiae* extracts (12). We adapted a similar approach to examine *cpc-1* uORF1 and uORF2 in *N. crassa* extracts. Adding cycloheximide (CYH) to reaction mixtures at time zero (*T*_0_) allows toeprint mapping of initiation codons where 80S ribosomes first initiate translation following initial scanning. Adding CYH at 10 min of incubation of translation reaction mixtures (*T*_10_) allows toeprint mapping of initiation sites in the steady state, for example, at additional sites where ribosomes have reinitiated. At *T*_0_ and *T*_10_, ribosomes are seen at the uORF1 AUG start codon; mutation to AAA eliminated this signal (Fig. 2B). This is expected since the uORF1 AUG is in an optimal initiation context. At *T*_0_, a reduced toeprint signal is seen at the uORF2 AUG relative to the signal at the uORF1 AUG. When the uORF1 AUG is mutated, the uORF2 AUG signal increased substantially; mutation of the uORF2 AUG to ACA eliminated this signal. These data indicate that most ribosomes initiate at uORF1 but, when it is absent, they scan to uORF2. At *T*_0_, a relatively low signal was observed at the mORF AUG except when uORF1 and uORF2 AUGs were mutated, as expected from scanning. When CYH was added at *T*_10_, the most dramatic change in signal was an increase at the mAUG in the ΔuORF2 construct. This increase of the mAUG was not seen in the ΔuORF1 construct or the ΔuORF1 ΔuORF2 construct. These data are consistent with ribosomes reinitiating at the mAUG following uORF1 translation *in vitro*. They suggest that uORF1 and uORF2 of *N. crassa cpc-1* function similarly to uORF1 and uORF4, respectively, of *S. cerevisiae GCN4*.

We next compared luciferase activities obtained from constructs with and without the introduced in-frame UAA stop codon to examine translation from NCCs upstream of the mAUG (Fig. 2C). The production of luciferase decreased when the UAA was present, indicating that polypeptides with luciferase activity were produced using NCCs upstream of the mORF (compare constructs 1 and 3, constructs 2 and 4, and constructs 3 and 6 in Fig. 2C). The UAA mutation decreased luciferase synthesis in the presence or absence of uORF2 (compare constructs 1 and 2 and constructs 3 and 4 [Fig. 2C]). Elimination of uORF2 resulted in overall increased luciferase synthesis as expected from its proposed inhibitory role for initiation at mAUG. As expected, NCCs and the mAUG have separate roles in initiation; combining ΔmAUG and UAA mutations yielded no detectable luciferase (Fig. 2C).

Interestingly, ΔmAUG showed a relatively small decrease in luciferase activity compared to WT (ΔmAUG, 71% of WT; compare constructs 1 and 5, Fig. 2C). This observation is consistent with the differences between the WT and the UAA constructs (UAA, 22% of WT; compare constructs 1 and 3, Fig. 2C). These data indicate that upstream NCCs are used to initiate translation efficiently *in vitro*. While this is the case, we did not identify NCCs by toeprint analyses (Fig. S7). Possibly, translation initiation is distributed among multiple NCCs, reducing signals at individual NCCs.

10.1128/mBio.00844-17.7 FIG S7  Toeprint analysis indicates reinitiation following translation of *cpc-1* uORF1 but not uORF2 (primary data for Fig. 2B). The description of the experiment is given in the text. For markers, either primer CPC101 (upper panel) or ZW4 (lower panel) was used to sequence the WT template (the four leftmost lanes). The nucleotide complementary to the dideoxynucleotide added to each sequencing reaction for the wild-type *cpc-1–luc* template is indicated above the corresponding lane so that the sequence of the template can be directly deduced; the 5′-to-3′ sequence reads from top to bottom. Asterisks (top to bottom), toeprint products corresponding to ribosomes bound at uAUG1, uAUG2, and mAUG on the *cpc-1–luc* RNA. Boxes (top to bottom), uAUG1, uAUG2, and mAUG in the sequence markers. The ribosome protects approximately 16 nt 3′ of a start codon in the ribosome P-site. Download FIG S7, PDF file, 0.3 MB. Copyright © 2017 Ivanov et al. 2017 Ivanov et al. This content is distributed under the terms of the Creative Commons Attribution 4.0 International license.

**FIG 3:**
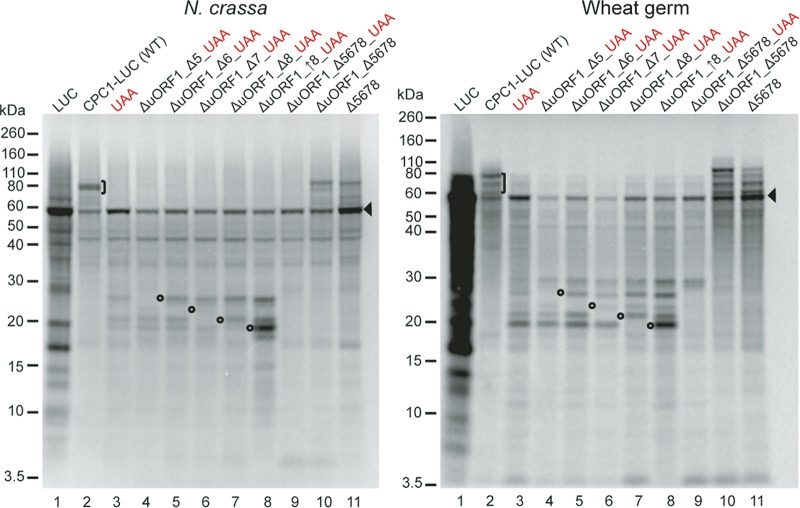
Evidence that *N. crassa* NCC5 to NCC8 initiate translation *in vitro*. Synthetic RNAs (60 ng) for the indicated constructs were used to program 10 µl of cell-free translation systems from *N. crassa* (left) or wheat germ (right). Reaction mixtures were incubated for 30 min at 26°C. Radiolabeled products were analyzed on 12% NuPAGE gels. Open circles, translation products eliminated upon mutation of NCC5 to NCC8; the product predicted to be initiated from NCC8 also increased when NCC8 was changed to AUG (lane 8). Arrowhead, position of mAUG-initiated translation product (mORF). Brackets, translation products larger than the mORF produced in the absence of an in-frame UAA stop codon.

The eight NCCs identified bioinformatically (Fig. S6) were individually eliminated, and the consequences were examined by [^35^S]Met labeling in *N. crassa* and wheat germ extracts (Fig. 3 and S7). These mutations were also combined with the UAA mutation so that the resulting polypeptides produced from upstream initiation could be better resolved by SDS-PAGE. Elimination of NCC1, NCC2, NCC3, or NCC4 did not yield any detectable differences compared to UAA (Fig. S8, lanes 3 to 7). In contrast, elimination of NCC5, -6, -7, or -8 resulted in disappearance of specific truncated polypeptides (Fig. S8, lanes 8 to 11 and 3, and Fig. 3, lanes 8 to 11 and 3), indicating that NCC5 to NCC8 initiated translation in *N. crassa* and wheat germ systems. When NCC8 (ACG) was changed to AUG, the signal in the corresponding band increased as expected (lanes 12 and 3, Fig. S8, and lanes 8 and 3, Fig. 3).

10.1128/mBio.00844-17.8 FIG S8  Evidence from [^35^S]Met labeling showing that NCC5 to -8, but not NCC1 to -4, initiate translation in an *N. crassa* cell-free system. Synthetic RNAs (60 ng) for the indicated constructs were used to program 10 µl of cell-free translation reaction mixtures from *N. crassa*. Reaction mixtures were incubated for 30 min at 26°C. Radiolabeled products were analyzed on 12% NuPAGE gels. Open circles, translation products eliminated upon mutation of NCC5 to -8; the product predicted to be initiated from NCC8 also increased when NCC8 was changed to AUG (lane 12). Arrowhead, position of mAUG-initiated translation product (mORF). Brackets, translation products larger than the mORF produced in the absence of an in-frame UAA stop codon. Download FIG S8, PDF file, 0.1 MB. Copyright © 2017 Ivanov et al. 2017 Ivanov et al. This content is distributed under the terms of the Creative Commons Attribution 4.0 International license.

Cell translation extracts programmed with CPC1-LUC (WT) produce polypeptides migrating more slowly than luciferase synthesized from an mRNA specifying LUC alone or a CPC1-LUC mRNA with the UAA mutation (Fig. 1A, 3, and S8). When NCC5, NCC6, NCC7, and NCC8 were eliminated together in the absence of the UAA mutation, polypeptides larger than luciferase were still observed, although the amount was reduced compared to WT (Fig. 3, compare lanes 10, 11, and 3). This suggests that other upstream codons in the *cpc-1* upstream region can be used to initiate polypeptide synthesis. This was observed in the presence or absence of uORF1 (lanes 10 and 11, Fig. 3).

**FIG 4:**
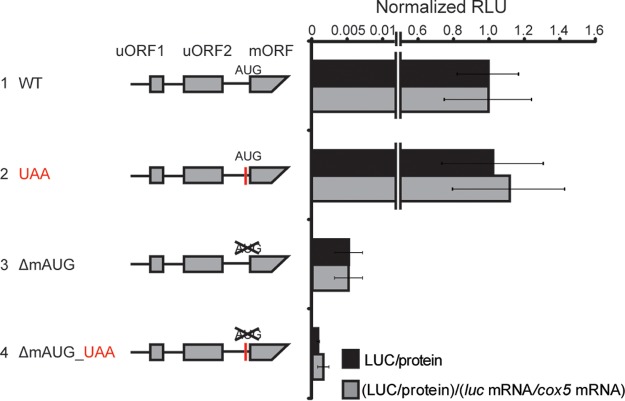
Discriminating translation from *N. crassa cpc-1* NCCs and mAUG *in vivo*. Constructs 1 to 4 were placed at the *N. crassa his-3* locus (three independent transformants of each). LUC activities were measured, and values were plotted relative to WT. Black bars, LUC activities normalized to total extracted protein; gray bars, LUC activities normalized first to total protein and then to *luc* mRNA/*cox-5* mRNA levels. Mean values and standard deviations for all measurements are derived from three independent experiments, each using all independent transformants.

To examine the roles of upstream NCCs in translation *in vivo* in *N. crassa*, strains containing *N. crassa* codon-optimized luciferase fused in frame with wild-type or mutated *cpc-1* 5′ sequences were constructed. Three independent transformants containing each construct were used to measure LUC activity and LUC mRNA levels. We examined WT, UAA, and ΔmAUG strains and the ΔmAUG UAA double mutant. Luciferase activity was measured and normalized to reporter mRNA levels to account for the small differences in luciferase mRNA levels observed. Expression levels of WT and UAA reporters were similar (in Fig. 4). Luciferase activity from the ΔmAUG construct was much lower, but this activity was higher than that for the ΔmAUG UAA construct. For the ΔmAUG construct, higher luciferase activity was observed than for the ΔmAUG UAA double mutant. Thus, although the amount of luciferase activity derived from upstream NCCs was less than 1% of activity from the mAUG *in vivo*, detectable luciferase was nevertheless observed (compare constructs 3, 1, and 2 in Fig. 4 and compare constructs 5, 1, and 3 in Fig. 2C). Possibly, NCCs were not used as efficiently *in vivo* as *in vitro*. Alternatively, N-terminally extended luciferases are less stable or less active *in vivo*, but we have not investigated this further.

**FIG 5:**
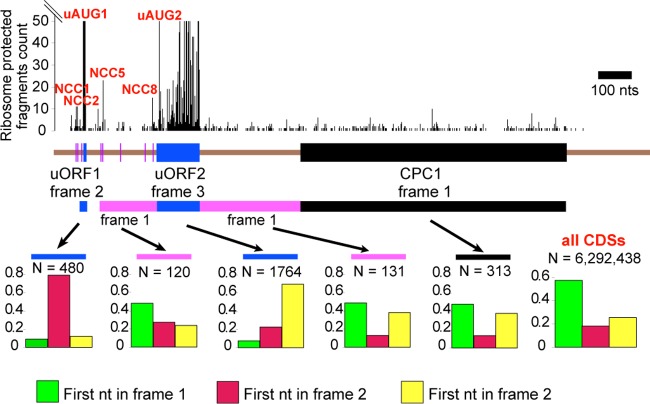
Ribosome profiling evidence for translation of the *cpc-1* N-terminal extension. The ribosome-protected fragment count (cutoff at 50) for each position 5′ to 3′ along the *cpc-1* mRNA is shown on top. Peaks that can be assigned to specific initiation codons, as labeled in Fig. 1B and K, are indicated in red. A schematic map of the ORF organization of *cpc-1*, drawn to scale, is shown below the ribosome-protected fragment count. The uORFs are represented by blue rectangles. The eight in-frame NCCs upstream of uORF2 are represented by magenta bars. The mORF is represented by a black rectangle. The reading frame of each feature relative to the mORF is indicated. The reading frame information derived from the ribosome-protected fragments for 5 specific regions along the *cpc-1* mRNA, as well as the tabulated data for all *N. crassa* coding sequences (CDSs), is shown at the bottom. The tabulation is done by scoring the first nucleotide of each ribosome-protected fragment relative to the reading frame of the annotated coding sequence (first to last ribosome A-site codons), using a 15-nucleotide 5′ offset—the proportions of total fragments mapped to frame 1 are shown by green bars, those mapped to frame 2 are shown by red bars, and those mapped to frame 3 are shown by yellow bars. The number of reads used to tabulate the data in each histogram is indicated as “N.”

For further investigation of translational activity in the region of *cpc-1* mRNA upstream of mAUG, data from ribosome profiling experiments in *N. crassa* were examined. Ribosome profiling provides snapshots of genome-wide *in vivo* translation by deep sequencing which is amenable to quantification. Cells were grown for 24 h in the dark, and ribosome profiling data were collected and analyzed as described in Materials and Methods. As shown previously, ribosome footprint data can be used to determine the frame in which a particular region of mRNA is being translated (29, 37, 38). The ribosome footprints for the *cpc-1* transcript (Fig. 5) show that uORF1 and uORF2 are heavily translated under these conditions. The frame information obtained with protected fragments of at least 28 nt using a 15-nt offset to the ribosome A site agrees with the predictions that, relative to the CPC1 frame, uORF1 is in frame 2 and uORF2 is in frame 3. The main CPC1 coding region contains ribosomes in the predicted reading frame (frame 1) as expected. Importantly, ribosome footprints in the 5′ region of the transcript outside uORF1, uORF2, and CPC1, especially between uORF2 and CPC1, were all in the CPC1 frame. This is consistent with the *in vitro* data showing in-frame translation upstream of the main CPC1 coding region. Furthermore, ribosome footprint data, with certain preparation protocols, show accumulation of footprints at AUG and non-AUG start codons (20, 29). This is also the case in the data set that was used for the present analysis of *N. crassa*. Accumulated footprints were observed at uAUG1 and uAUG2 and at four of the eight predicted NCCs (Fig. 5).

As controls, we examined ribosomes in the 5′ leaders of *arg-2* (NCU07732) and *eif5* (NCU00366), which are two other *N. crassa* mRNA transcripts that contain uORFs (31, 39). In addition to the ribosome footprints in the main ORFs that preferentially corresponded to the predicted reading frame, ribosomes are observed in the uORFs (Fig. S9). The frame information of the footprints in the main ORFs of the uORFs matches the predictions based on the gene model of the corresponding mRNAs. The footprints in the 5′ leaders outside the uORFs appear not to be spurious. Positions represented by more than 2 footprints correspond to near-cognate start codons. For example, each of the six larger peaks 5′ of the first uORF of eIF5 precisely (i.e., with 1-nt resolution) matches six NCCs—AUC, UUG, CUG, GUG, UUG, and UUG, respectively.

10.1128/mBio.00844-17.9 FIG S9  Ribosome profiling data, including framing information, for *arg-2* and *eif5*. The ribosome-protected fragment count for each position 5′ to 3′ along the mRNA is shown on top for *arg-2* (NCU07732) (A) and *eif5* (NCU00366) (B). Peaks corresponding to specific NCCs in the 5′ leader of *eif5* are shown by red arrows. A schematic map of the ORF organization, drawn to scale, is shown below the ribosome-protected fragment count graph. The reading frame information derived from the ribosome-protected fragments for specific regions along the mRNAs is shown below the schematic maps. AAP, uORF-encoded arginine attenuator peptide; all CDSs, all predicted *N. crassa* genic ORFs. The color coding for different components of the figure is the same as in Fig. 5. Download FIG S9, PDF file, 0.2 MB. Copyright © 2017 Ivanov et al. 2017 Ivanov et al. This content is distributed under the terms of the Creative Commons Attribution 4.0 International license.

Although the ribosome profiling experiment described above provided strong evidence for *in vivo* translation of the region upstream of the mAUG in the CPC1 frame, it did not and could not address the question of whether translation of this region increases or decreases under stress conditions. The answer is important to address the question of whether the N-terminal extension is part of the translational regulation of *cpc-1* or whether it merely provides a constitutive alternative isoform of *cpc-1*. To address this question, a second ribosome profiling experiment was performed. In it, *N. crassa* cells were grown in the presence or absence of 3-amino-1,2,4-triazole (3AT), which induces histidine starvation. Compared to untreated cells, as expected, 3AT cells showed elevated density in the mORF compared to uORF2, with the ratio of the ribosome footprint counts in the two regions changing from 0.57 in untreated cells to 1.66 in 3AT-treated cells. However, the ratio of the ribosome footprint count in the region between uORF2 and the mORF relative to the footprint count in the mORF remains nearly unchanged—0.19 in untreated cells to 0.21 in 3AT-treated cells. This result is consistent with the idea that amino acid starvation induces translation at the non-AUG codons of *cpc-1* responsible for initiation of the N-terminal extension. Consistent with this notion, the ratio of ribosome footprint count in the region between uORF1 and uORF2, where non-AUG initiation of the N-terminal extension must occur, to the footprint count in uORF2 increases in 3AT-treated cells compared to control cells—from 0.11 to 0.24.

## DISCUSSION

We examined the structures of *N. crassa cpc-1* homologs in fungi for which sequence was available. In Pezizomycotina, all *cpc-1* genes specify two uORFs, uORF1 and uORF2, within an extended mRNA 5′ leader. The data obtained here with *N. crassa* are consistent with uORF1 and uORF2 functioning analogously to *S. cerevisiae GCN4* uORF1 and uORF4, respectively, to control initiation at the predicted mAUG start codon. Surprisingly, a long, conserved coding region upstream of this AUG start codon that was in frame with CPC1 was present in all homologs from Pezizomycotina, and in some cases, this open reading frame extended to the predicted mRNA 5′ ends. While no AUG codons were observed that could produce N-terminally extended isoforms of CPC1 (excepting the possibility of ribosomal frameshifting from a uORF AUG), near-cognate start codons (NCCs), some well conserved, were present in the CPC1 reading frame that potentially could initiate translation of such isoforms. Translation initiating from four conserved NCCs in the *N. crassa cpc-1* 5′ leader was observed *in vitro* in *N. crassa* and wheat germ translation extracts. Utilization of NCCs *in vivo* would result in synthesis of alternative isoforms of CPC1; these isoforms may have similar or different functions than CPC1 produced from the main AUG. N-terminal extensions could also influence protein stability. Only future experiments can distinguish between these possibilities. The synthesis of these alternative isoforms from NCCs upstream of uORF2 would also bypass the inhibitory effect of uORF2, which reduces synthesis of CPC1 from the downstream main AUG. These findings suggest a model for additional translational regulation of Pezizomycotina *cpc-1* through the use of NCCs, which could be independent of the uORF control model elucidated for *S. cerevisiae GCN4*. Another potential mechanism that could contribute to translation in the CPC1 reading frame upstream of the main AUG that is also consistent with these data is +1 (or −2) translational frameshifting occurring within uORF2, since all uORF2s in Pezizomycotina analyzed thus far are in the −1 frame relative to the mORF.

We found no fungal homologs of *cpc-1/GCN4* outside Pezizomycotina and Basidiomycota that have NCC-initiated N-terminal extensions with the potential to preempt the effect of translating a long and inhibitory uORF. Since the other two subphyla of Ascomycota, Saccharomycotina and Taphrinomycotina, do not have potential for NCC-initiated extensions, it not entirely clear if the conserved extensions present in Pezizomycotina and in Basidiomycota (a sister phylum of Ascomycota in the subkingdom Dikarya) are homologous and were present in the last common ancestor of Dikarya, which lived around 500 million years ago (33), or whether they are examples of convergent evolution.

In the studies reported here, there is a discrepancy between luciferase activities produced *in vitro* and those produced *in vivo* from the *N. crassa cpc-1* NCCs. At face value, this would mean that *in vitro* there is more initiation from NCCs than from the mAUG, while *in vivo* the situation is reversed. For the *in vitro* experiments, we used an intermediate [Mg^2+^], which favors AUG over NCC initiation, but the *in vitro* conditions used here are not expected to be as stringent as *in vivo* (17). Thus, we expect that relatively more NCC-initiated products would be produced *in vitro* than *in vivo*, but the discrepancy in levels of CPC1-LUC activity observed still seems too large to be simply accounted for by this consideration, given that the ribosome profiling data support translation from NCCs *in vivo*. It is possible that the N-terminally extended forms of the luciferase reporter are unstable *in vivo* and that luciferase reporter data might thus provide accurate information on the relative level of N-terminally extended CPC1 isoforms *in vivo*. This level, while low, could nevertheless be physiologically significant. This conclusion is further strengthened by the ribosome profiling data. It too suggests that under normal conditions translation of the mORF, though low, is primarily initiated upstream of the stop codon of uORF2 (e.g., at NCCs). Taken together, these data raise important new questions regarding the functions of the isoforms of CPC1 and their regulation.

The results from ribosome profiling following 3AT treatment raise several intriguing questions regarding the likelihood that the NCCs in *cpc-1* are used for regulation and also about the nature of this regulation. The standard model of *cpc-1* regulation under amino acid limitation posits that eIF2 phosphorylation reduces translation of the inhibitory uORF2 by lengthening the time of reinitiation. Yet, total translation of the region between uORF1 and uORF2 appears to increase following 3AT treatment. Either NCC initiation becomes very efficient under amino acid limitation in general, overcoming the inhibitory effect of reduced reinitiation, or translation of uORF1 specifically primes retained ribosomes for initiation at the NCC in response to amino acid limitation.

CPC1 is a bZIP transcription factor, and the mammalian bZIP transcription factor family of CCAAT/enhancer binding proteins (C/EBPs) provides potential context for how bZIP isoforms are produced by alternative initiation to have different functions. C/EBPα initiates at an in-frame AUG in a poor initiation context; C/EBPβ initiates from an NCC in a good context (40). Leaky scanning past the latter initiation codon leads to initiation at an out-of-frame AUG codon in good context, producing a short ORF. Two additional C/EBP isoforms (LAP and LIP) are generated by initiation from in-frame AUG codons downstream of this short ORF by reinitiation following translation of the short ORF, and the relative levels of LAP and LIP can be altered by changes in eIF2α phosphorylation. LAP functions as a transcriptional activator and LIP functions as a transcriptional repressor, modulating different transcriptional outcomes under “normal” and stress conditions.

It is worth considering that the translation of another fungal bZIP transcription factor, *Podospora anserina* IDI-4, is proposed to initiate from a CUG and not an AUG codon, and this CUG is conserved in the *N. crassa* homolog (41). Thus, possibly, fungal bZIP transcriptional factors may more generally use NCCs to initiate their translation.

The physiological conditions that govern initiation at NCCs are an emerging area of investigation, and the evolutionarily conserved features in the 5′ UTRs of filamentous fungal CPC1 homologs provide an additional new architecture to confer 5′ UTR translation regulation (42). In *S. cerevisiae*, amino acid limitation increases initiation at NCCs (29), as does the shift to the meiotic developmental program (43), at least for genes other than *GCN4*. A chemical screen identified several compounds that increase the efficiency of initiation at NCCs (44). The concentration of free polyamines affects initiation from a conserved AUU start codon of a uORF within the mRNA encoding AZIN1 in mammalian cells (45). A number of cellular factors are known to be involved in discrimination between favorable and unfavorable initiation codons and contexts (46–49). Changes in the activity or cellular levels of eIF1 or eIF5 can have profound effects on translation initiation at NCCs or AUG codons in a poor context (9, 30, 31, 50–52). Understanding the physiological conditions that control initiation at NCCs has broad implication for gene regulation and protein synthesis as well as for specific understanding of these aspects of CPC1.

## MATERIALS AND METHODS

### Sequence assembly and analysis.

All *cpc-1* sequences were obtained from GenBank by BLAST with the *N. crassa cpc-1* sequence as the starting point. In most cases, the sequences were derived from the whole-genome shotgun contigs (WGS) database. WGS sequences were processed manually to predict intron/exon junctions for the mRNA sequence. In a minority of cases, sequences were available from expressed sequence tags (ESTs). EST data were manually assembled into contigs. Additional sequences were obtained from the transcriptome shotgun assembly (TSA) database. All alignments in this study were performed with the ClustalX2 and ClustalW algorithms. Sequences used in this study are available upon request.

Maximal (stop-codon-to-stop-codon) *cpc-1* ORFs from 96 fungal species were translated and aligned as amino acids with MUSCLE (53), and the amino acid alignment was used to guide a codon-based nucleotide alignment (EMBOSS tranalign [54]). The alignment was mapped to *N. crassa* coordinates by removing all alignment columns that contained a gap character in the *N. crassa* sequence and analyzed with the codonml program in the PAML package (36), synplot2 (55) using a 5-codon sliding window, and MLOGD (35) using a 20-codon sliding window and 1-codon step size. For MLOGD, the null model in each window is that the sequence is noncoding while the alternative model is that the sequence is coding in the given reading frame. Standard deviations for the codonml *dN/dS* values were estimated via a bootstrapping procedure, in which codon columns of the alignment were randomly resampled (with replacement); 100 randomized alignments were generated for each region, and their *dN/dS* values were calculated with codonml.

### Plasmids.

The starting point for all constructs was plasmid pPC01 (Z. Wang and M. Sachs, unpublished data), which has the 5′ leader of *N. crassa cpc-1* cloned between BamHI and XhoI sites (the latter located at the 5′ end of the firefly luciferase cassette). First, the sequence GTCTTC, just upstream of the NCC8 ACG codon in the 5′ leader, was changed by two-step PCR to a SacI GAGCTC sequence to facilitate making subsequent mutations. This derivative is named pPC100 and is referred to as wild type (WT).

Specifics about plasmids are provided in Table S1A and B in the supplemental material. For *in vitro* experiments, pPC-series plasmids with the luciferase gene (not codon optimized) were used. When two PCR primers are shown in a cell in Table S1A, one-step PCR was used to generate inserted regions from corresponding PCR templates. When four PCR primers are shown, two-step PCR was used to generate inserted regions. PCR products and vectors were digested by restriction enzymes, gel purified, and ligated. For pPC176, synthetic complementary oligonucleotides were annealed and ligated to gel-purified vector pPC100 that had been digested with AgeI and XhoI. For *in vivo* assay mixtures that contained codon-optimized luciferase, plasmids pJI500, pJI502, pJI501, and pJI576 were made by replacing the small BamHI-NsiI fragment of pJI401 with the small BamHI-NsiI fragments of pPC100, pPC102, pPC101, and pPC176, respectively.

10.1128/mBio.00844-17.10 TABLE S1  Plasmids (A) and primers (B) used in the study. Download TABLE S1, PDF file, 0.1 MB. Copyright © 2017 Ivanov et al. 2017 Ivanov et al. This content is distributed under the terms of the Creative Commons Attribution 4.0 International license.

### RNA synthesis and cell-free translation.

Capped and polyadenylated RNAs were transcribed *in vitro* by T7 RNA polymerase from plasmid DNA templates that were linearized with EcoRI, and the relative amounts of RNA were determined as described previously (17). *In vitro* translation and gel analysis for visualizing ^35^Met-labeled proteins using *N. crassa* extracts and wheat germ extract were accomplished as described previously (17), except that 10 µl of translation reaction mixtures was incubated for 30 min at 25°C and samples were mixed with 10 μl 2× NuPAGE LDS sample buffer (Invitrogen) and put on ice to stop reactions. *In vitro* translation for luciferase activity assays using *N. crassa* extracts was accomplished as described previously (17) using 6 ng of each mRNA to program extracts. Primer extension inhibition (toeprint) assays were accomplished using ^32^P-labeled primers CPC101 and ZW4 as described previously (17), except that 0.5 mg/ml cycloheximide was added to the reaction mixtures as indicated in Results.

### Strains, culture conditions, and *in vivo* measurements.

Strain FGSC 6103 [*his-3* (Y234M723) *mat A*] and the wild-type (WT) reference strain FGSC 2489 (74-OR23-1V *mat A*) were obtained from the Fungal Genetics Stock Center (FGSC) (56). Targeting of firefly luciferase reporters to the *N. crassa his-3* locus by transformation of FGSC 6103 with PciI-linearized plasmid DNA (pJI500, pJI501, pJI502, or pJI576), culture conditions, and conditions for luciferase assays were as described previously (17). Total RNA was prepared from cells, cDNA was prepared, and reverse transcription-quantitative PCR (RT-qPCR) was performed as described previously (17).

### Ribosome profiling.

*N. crassa* cultures were grown for 24 h in the dark, and following the breakage procedure used for the preparation of *N. crassa* cell translation extracts (57), but using the buffers described previously (58). Ribosome-protected mRNA fragments were prepared for sequencing essentially as described previously (58), except that 50 *A*_260_ units of lysate was used, nucleic acid pellets recovered from each step were washed with 80% ethanol, and the rRNA depletion step was omitted. Libraries were sequenced on an Illumina HiSeq 2000 sequencer, generating 54,446,346 51-mer reads after removal of multiplexing adapter sequences. Reads were further trimmed to remove the CTGTAGGCACCATCAAT adapter sequence with cutadapt-1.2.1 (options -n 1 -m 28) (59). Trimmed reads of 28 to 31 nt in length were mapped to *Neurospora* transcripts with Bowtie version 0.12.9 (options -n 0 -l 25 -a –norc) (60). Counts of reads at each framing position were generated with Python scripts as described previously (38).

### Accession number(s).

Sequences of ribosome profiling (Ribo-Seq) libraries have been deposited in the NCBI Genome Expression Omnibus (GEO) under accession number GSE97717.
