# Supplementary material for: Translation Initiation from Conserved Non-AUG Codons Provides Additional Layers of Regulation and Coding Capacity
Source: mBio. 2017 Jun 27;8(3):e00844-17. doi: 10.1128/mBio.00844-17 (PMC5487733; doi:10.1128/mBio.00844-17)
Supplement: FIG S1 [file mbo003173356sf1.pdf]

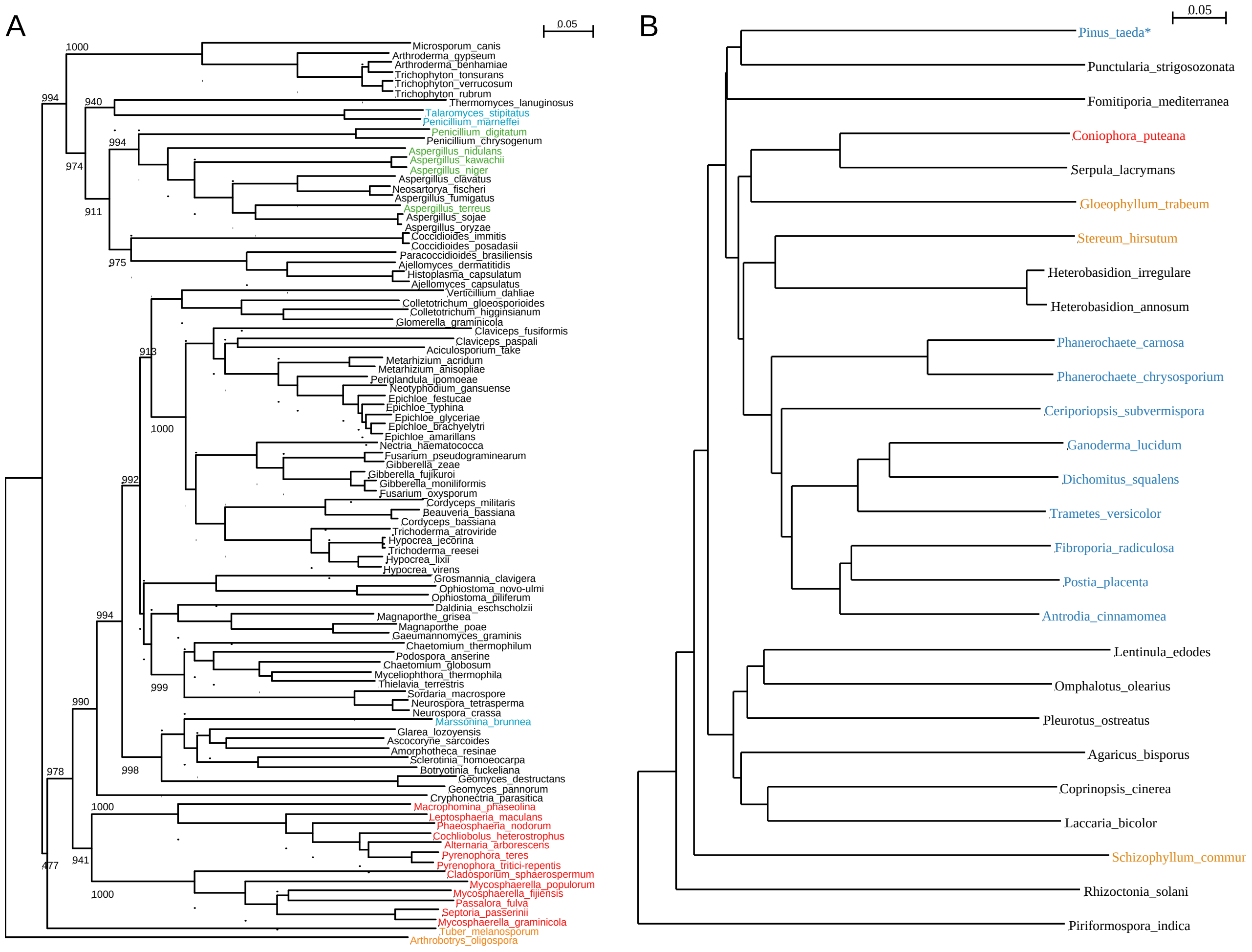

Supplementary Figure 1. Superimposing conservation of NCCs in cpc-1 homologs on the phylogenetic trees of Pezizomycotina and Agaricomycetes. (A) Phylogeny of Pezizomycotina based on CPC1 amino acid sequences and conservation of NCCs 6, 7 and 8. The unrooted phylogenetic tree is based on the amino acid sequences (from the 5' end to the mORF stop codon when no in-frame stop codon is present, or from the stop codon in the 5' region to the mORF stop codon) of the *cpc-1* homologs from the Pezizomycotina species indicated on the right of each branch. Species names are coded with respect to which NCCs are identical to *N. crassa*: black, NCCs 6, 7 and 8; blue: 7 and 8; green: 6 and 8; red: 8; orange: no identical NCCs. (B) Phylogeny of Agaricomycetes based on CPC1 amino acid sequences and conservation of a single NCC. The unrooted phylogenetic tree is based on the amino acid sequences (from the 5' end to the mORF stop codon, or from the stop codon in the 5' region to the mORF stop codon) of the *cpc-1* homologs from the Agaricomycetes species indicated on the right of each branch. The asterisk indicates a sequence presumed to derive from an unknown Agaricomycetes species contaminating the *Pinus taeda* EST library. Species names in black indicate NCC = AUU; names in blue, NCC = UUG; in orange, NCC = AUA; in red, NCC = ACG or CUG. The trees were generated using ClustalX. Bootstrap values are given for key nodes in Pezizomycotina are shown. The scale represents divergence rate at each residue.
