# Supplementary material for: Translation Initiation from Conserved Non-AUG Codons Provides Additional Layers of Regulation and Coding Capacity
Source: mBio. 2017 Jun 27;8(3):e00844-17. doi: 10.1128/mBio.00844-17 (PMC5487733; doi:10.1128/mBio.00844-17)
Supplement: FIG S2 [file mbo003173356sf2.pdf]

A

Species name	uAUG1	uAUG2	mAUG	NCC6	NCC7	NCC8
Aciculosporium take	GUCAAGAUGC	AUCACCAUGG	UUCAAGAUGA	GUUGAUCUGG	GCCAACAUUU	UUCACGACGG
Ajellomyces capsulatus	AAGAUUAUGG	GUCACCAUGG	UUGAAUAUGC	GGGCCUCUGA	ACAGCCAUUU	UUCACCACGG
Ajellomyces dermatitidis	CAAAUUAUGG	GUCAACAUGG	CGUAUUAUGU	GGAUCUCUGA	CCAAACAUUU	UUCACCACGG
Alternaria arborescens	CGAACAAUGG	GUCAUCAUGG	ACAAACAUGG			ACCAUCACGG
Alternaria brassicicola	CGAACAAUGG	GUCAUCAUGG	ACAAACAUGG			ACCAUCACGG
Amorphotheca resinae	UCCAAGAUGU	ACCACCAUGG	CCCAACAUGG	GAAAACCUGA	GCCACAAUAC	UUCAACACGG
Arthrobotrys oligospora	GCCAAAAUGG	GCCAGGAUGG	GUCGAUAUGG			
Arthroderma benhamiae	UACAAGAUGG	CCCAUCAUGG	GCCGCAAUGG	GGACCUCUGA	ACCACCAUUC	UUCACCACGG
Arthroderma gypseum	UACAAGAUGG	CCCAUCAUGG	GCCGCUAUGG	GGACCUCUGA	ACCACUAUUU	UUCACCACGG
Ascocoryne sarcoides	GCCACGAUGU	ACCAAGAUGG	CCAAACAUGG	GAAUCUCUGA	GCCAACAUAC	UUCACCACGG
Aspergillus clavatus	UGCAAAAUGG	GCAAUCAUGG	CGCAUCAUGU	GGACCUCUGA	AACAAUAUUC	UUCACCACGG
Aspergillus fumigatus	UGCAAAAUGG	GCAAACAUGG	CGCAUCAUGU	GGACCUCUGA	AACAACAUUC	UUCACCACGG
Aspergillus kawachii	UUCAAAAUGG	GCAAUCAUGG	CGCAUCAUGU	GGACCUCUGA		UUCACCACGG
Aspergillus nidulans	CUCAAAAUGG	GCAAUCAUGG	CGCAACAUGU	GAACCUCUGA		UUCACCACGG
Aspergillus niger	UUCAAAAUGG	GCAAUCAUGG	CGCAUCAUGU	GGACCUCUGA		UUCACCACGG
Aspergillus oryzae	UCCACAAUGG	GCAAACAUGG	CGCAUAAUGU	GGACCUCUGA	AACAACAUUU	UUCAACACGG
Aspergillus sojae	UCCAAAAUGG	GCAAACAUGG	CGCAUAAUGU	GGACCUCUGA	AACAACAUUU	UUCAACACGG
Aspergillus terreus	CCCAAAAUGA	GCAAUCAUGG	CGCAUCAUGU	GGACCUCUGA		UUCACCACGG
Beauveria bassiana	UUCAAGAUGC	GUCAUCAUGG	GCCACGAUGA	GUCGACCUGA	GCCAACAUUU	UUCACCACGG
Botryotinia fuckeliana	UUCAACAUGU	AUCACCAUGG	CCAAACAUGG	GAAAAUCUGA	GCAACAAUAC	UUCACCACGG
Chaetomium globosum	UUCAAGAUGC	GUCAACAUGG	GCCAAGAUGA	GUUGAACUGA	GCCGCCAUUC	UUCACCACGG
Chaetomium thermophilum	UUCAAAAUGC	GUCACCAUGG	GCCAAGAUGA	GUUGAACUGA	GCCACAAUUC	UUCACCACGG
Cladosporium sphaerospermum	GACACCAUGG	GCAAUCAUGG	AACACCAUGG			UCCACCACGG
Claviceps fusiformis	GCCAAGAUGU	GUCAACAUGG	GCCAAUAUGA	GUUGAACUGG	GCCAACAUUU	UUCACCACGG
Claviceps paspali	GCCAAGAUGU	GUCAACAUGG	GCGAAGAUGA	GCUGACCUGG	GCCAAUAUUC	UUCGUCACGG
Coccidioides immitis	UCCAUCAUGA	GUCAACAUGG	CGAGCGAUGU	GGACCUCUGA	CCAACCAUCA	UUCACCACGG
Coccidioides posadasii	UCCAUCAUGA	GUCAACAUGG	CGAGCGAUGU	GGACCUCUGA	CCAACCAUCA	UUCACCACGG
Cochliobolus heterostrophus	AUCAUAAUGG	GUCAACAUGG	ACAAACAUGG			UCCACCACGG
Colletotrichum gloeosporioides	CUCAAGAUGC	AUCACCAUGG	UCAAAGAUGG	GUCGAUCUGA	UCCACCAUUU	UUCACCACGG
Colletotrichum higginsianum	GCAACCAUGC	AUCAUCAUGG	CCAAACAUGG	GUCGAUCUGA	GCAACGAUUC	CUCACCACGG
Cordyceps militaris	UUCAAGAUGG	GUCAACAUGG	GGCACGAUGA	GUCGACCUGA	GCCAACAUUU	UUCACCACGG
Cryphonectria parasitica	AUCAAGAUGU	GUCAACAUGG	AACAAUAUGG			GCCAACACGG
Daldinia eschscholzii	AUCAAAAUGC	GUCAAAAUGG	GCCAGGAUGG	GAAGAACUGA	GCCACCAUUC	UUCACCACGG
Epichloe amarillans	GUCAAGAUGC	AUCAACAUGG	GGCAAGAUGA	GUUGAUCUGG	GCCAAAAUUC	UUCACCACGG
Epichloe brachyelytri	GUCAAGAUGC	AUCAACAUGG	GGCAAGAUGA	GUUGAUCUGG	GCCAAAAUUC	UUCACCACGG
Epichloe festucae	GUCAAAAUGC	AUCAACAUGG	GGCAAGAUGA	GUUGAUCUGG	GCCAAAAUUC	UUCACCACGG
Epichloe glyceriae	GUCAAGAUGC	AUCAACAUGG	GCCAAGAUGA	GUUGAUCUGG	GCCAAAAUUC	UUCACCACGG
Epichloe typhina	GUCAAGAUGC	AUCAACAUGG	GGCAAGAUGA	GUUGAUCUGA	GCCAAAAUUC	UUCACCACGG
Fusarium oxysporum	GUCAAGAUGU	GUCACCAUGG	GCCAAGAUGA	GUCGACCUGA	GCCAACAUUU	UUCACCACGG
Fusarium pseudograminearum	AUCAAGAUGU	AUCACCAUGG	GCAAAGAUGA	GUCGACCUGA	GCCAACAUUU	UUCACCACGG
Fusarium sporotrichioides	AUCAACAUGU	AUCACCAUGG	GCAAAGAUGA	GUCGACCUGA	GCCAACAUUU	UUCACCACGG
Gaeumannomyces graminis	AUCAAGAUGU	GUCACCAUGG	GGCAAGAUGA	GAAGACCUGA	GUCACCAUUC	UUCACCACGG
Geomyces destructans	CCCAAAAUGG	GUCACUAUGG	ACCAACAUGC	CAAGACCUGA	UCCACCAUUC	UUUACGACGG
Geomyces pannorum	CACAAAAUGG	GUCAACAUGG	AACAACAUGG	GAAGAUCUGA	ACCACCAUUC	UUUACGACGG
Gibberella fujikuroi	GUCAAGAUGU	GUCACCAUGG	GCCAAGAUGA	GUCGAUCUGA	GCCAACAUUU	UUCACCACGG
Gibberella moniliformis	GUCAAGAUGU	GUCACCAUGG	GCCAAGAUGA	GUCGACCUGA	GCCAACAUUU	UUCACCACGG
Gibberella zeae	AUCAAGAUGU	AUCACCAUGG	GCAAAGAUGA	GUCGACCUGA	GCCAACAUUU	UUCACCACGG
Glarea lozoyensis	GACACGAUGU	ACCACCAUGG	CCAAACAUGG	GAAGAUUUGA	GCCACGAUAC	UUCACCACGG
Glomerella graminicola	GCAACCAUGC	AUCACAAUGG	CCCAAGAUGG	GUCGAUCUGA	GCAACCAUUC	UUCACCACGG
Grosmannia clavigera	GCAAAGAUGC	GUCAACAUGG	AGCAAAAUGG	GUCGAUCUGA	GCAACCAUUU	UUCAUCACGG
Histoplasma capsulatum	AAGAUUAUGG	GUCACCAUGG	UUGAAUAUGC	GGGCCUCUGA	ACAGCCAUUU	UUCACCACGG
Hypocrea lixii	UUCAAGAUGA	GUCAACAUGG	GCCAAGAUGA	GUCGAUCUGA	GCCAACAUUU	UUCACCACGG
Hypocrea virens	UUCAAGAUGA	GUCAACAUGG	GCCAAGAUGA	GUCGACCUGA	GCCAACAUUU	UUCACCACGG
Leptosphaeria maculans	AUCAAUAUGG	GACACCAUGG	ACAACUAUGG			UCCACCACGG
Macrophomina phaseolina	GUCAAAAUGU	GUCACCAUGG	GGCACCAUGA			UUCACCACGG
Magnaporthe grisea	GCCAAAAUGC	GUCACCAUGG	ACCAAGAUGA	CCAGCUCUGC	GCCGCCAUUC	UUCACCACGG
Magnaporthe poae	AUCAAGAUGU	GUCACCAUGG	GGCAAGAUGA	GAAGACCUGA	GUCACCAUUC	UUCACCACGG
Marssonina brunnea	UCCACCAUGU	CCCAAGAUGG	CCCAACAUGG		UUCGCAAUUC	GCCACCACGG
Metarhizium acridum	UUCAAGAUGC	AUCAACAUGG	GCCAAGAUGA	GUUGAUCUGG	GCCACCAUUU	UUCACCACGG
Metarhizium anisopliae	UUCAAGAUGC	AUCAACAUGG	GCCAAGAUGA	GUUGAUCUGG	GCCACCAUUU	UUCACCACGG
Microsporum canis	UACAAGAUGG	CCCAUCAUGG	CAAACUAUGG	GGACCUCUGA	UUCACCAUUC	UUCAUCACGG
Myceliophthora thermophila	ACCAAGAUGC	GUCAAGAUGG	GGCAAGAUGA	GUUGAACUGA	GCCGCCAUUC	UUCACCACGG
Mycosphaerella fijiensis	ACGAAAAUGG	GCAAACAUGG	UUCGCAAUGG			UCCACCACGG
Mycosphaerella graminicola	UUAAACAUGG	GCAAACAUGG	UUCGCAAUGG			UCCACGACGG
Mycosphaerella populorum	UUCAAGAUGU	GCAAACAUGG	GGCACUAUGG			CCCACCACGG
Nectria haematococca	GUCAAGAUGU	GUCAUCAUGG	GCCAAGAUGA	GUCGACCUGA	GCCAACAUUU	UCAACCACGG
Neosartorya fischeri	UGCAAAAUGG	GCAAACAUGG	CGCAUCAUGU	GGACCUCUGA	AACAACAUUC	UUCACCACGG
Neotyphodium gansuense	GUCAAGAUGC	AUCAACAUGG	GCCAAGAUGA	GUUGAUCUGG	GCCAAAAUUC	UUCACCACGG
Neurospora crassa	AUCAAGAUGC	GUCAACAUGG	AACAAGAUGU	GUAGAUCUGA	GCCACCAUUC	UUCACCACGG
Neurospora tetrasperma	AUCAAGAUGC	GUCAACAUGG	AACAAGAUGU	GUAGAUCUGA	GCCACCAUUC	UUCACCACGG
Ophiostoma novo-ulmi	GCCACAAUGC	GCAAACAUGG	AAAAAAAUGG	GUCGAUCUGA	GUCACAAUUC	UUCACAACGG
Ophiostoma piliferum	GACACCAUGC	GCAAACAUGG	ACCAACAUGG	GUCGAAUUGA	GUCACCAUUC	UUCACAACGG
Paracoccidioides brasiliensis	ACGACGAUGG	GCCACCAUGC	CGCAUCAUGU	GGACCUCUGG	GUCACCAUUC	UUCAACACGG
Passalora fulva	ACGAACAUGG	GCAAACAUGG	UUCGCGAUGG			UCCACCACGG
Penicillium chrysogenum	GCCAAAAUGG	GCAACCAUGG	CGUGUCAUGU	GGACCUCUGA	ACCCCGAUCG	UUCAACACGG
Penicillium digitatum	GCCAAAAUGG	GCAAACAUGG	CGUGUCAUGU	GGACCUCUGA		UUCACCACGG
Penicillium marneffei	UCCAUAAUGG	GCAAUCAUGG	AGAGUCAUGU		ACCACUAUUU	UUCACCACGG
Periglandula ipomoeae	GUCAAGAUGC	AUCAACAUGG	GCCAAGAUGA	GUUGAUCUGG	GCCAACAUUU	UUCAACACGG
Phaeosphaeria nodorum	AACAAAAUGG	GCAAAAAUGG	GCAGCCAUGG			UCCACCACGG
Podospora anserine	ACCAAGAUGC	GUCAUCAUGG	GGCAAGAUGA	GUUGAACUGA	ACCGCCAUUC	UUCACCACGG
Pyrenophora teres	CGAACAAUGG	GUCAUCAUGG	ACAAACAUGG			UCCAUCACGG
Pyrenophora tritici-repentis	CGAACAAUGG	GUCAUCAUGG	ACAAACAUGG			UCCAUCACGG
Sclerotinia homoeocarpa	AACAAGAUGU	AUCAACAUGG	CCAAACAUGG	GAAUCUUUGU	GCAACAAUAC	UUCACCACGG
Sclerotinia sclerotiorum	CUCAAAAUGU	AUCAACAUGG	AUAGACAUGG	GAAAAUCUGA		UUCACAACGG
Septoria passerinii	GUUAACAUGG	GCAAACAUGG	UUCGCAAUGG			UCCACGACGG
Sordaria macrospore	AUCAAGAUGC	GUCAACAUGG	AACAAGAUGU	GUAGAUCUGA	GCCACCAUUC	UUCACCACGG
Talaromyces stipitatus	UCCAUAAUGG	GCAAUCAUGG	AGAGUCAUGU		UCCACCAUUU	UUCACCACGG
Thermomyces lanuginosus	GUCAUAAUGG	GCAACCAUGG	CAAGUCAUGG	GAUUCGUUGA	AACAACAUCU	UCCACCACGG
Thielavia heterothallica	ACCAAGAUGC	GUCAAGAUGG	GGCAAGAUGA	GUUGAACUGA	GCCGCCAUUC	UUCACCACGG
Thielavia terrestris	AUCAAGAUGC	GUCAAGAUGG	GGCAAGAUGA	AGCACCCUGC	GCCGCCAUUC	UUCACCACGG
Trichoderma atroviride	UUCAUGAUGA	GUCAACAUGG	GCCAAGAUGA	GUCGACCUGA	GCGAACAUUU	UUCACCACGG
Trichoderma reesei	UUCAAGAUGA	GUCAACAUGG	GCCAAGAUGA	GUCGACCUGA	GCCAACAUUU	UUCACCACGG
Trichophyton rubrum	UGCAAGAUGG	CCCAUCAUGG	GCGGAUAUGG	GGACCUCUGA	AUCACCAUUC	UUCACCACGG
Trichophyton tonsurans	UACAAGAUGG	CCCAUCAUGG	GCCGCAAUGG	GGACCUCUGA	AUCACCAUUC	UUCACCACGG
Trichophyton verrucosum	UGCAAGAUGG	CCCAUCAUGG	GCGGAUAUGG	GGACCUCUGA	AUCACCAUUC	UUCACCACGG
Tuber melanosporum	GUUAUCAUGG	CCAGCCAUGG	UACAAAAUGC			
Verticillium dahliae	AUCACCAUGC	UACACCAUGG	GCGAACAUGA	GUUGAUCUGA	UUCUCCAUCA	GUCACCACGG
“Citrus clementina” #1	GACACCAUGG	GCAAUCAUGG	AUCACUAUGG			UCCACCACGG
“Citrus clementina” #2	GACAUCAUGG	GCAAUCAUGG	AUCACCAUGG			UCCACCACGG
“Malus x domestica”	AGAGUCAUGU	ACCAACAUGG	CCGACCAUGA			GCCACCACGG

B

```
Agaricus bisporus               GUAGCUAUUU
Antrodia cinnamomea             UCAGCCUUGU
Ceriporiopsis subvermispora     CCCGCCUUGU
Coniophora puteana              AGAACGCUGC
Coprinopsis cinerea             GGCGCCAUUU
Dichomitus squalens             CCCACCUUGU
Fibroporia radiculosa           UCCACCUUGU
Fomitiporia mediterranea        CCAGCCAUUC
Ganoderma lucidum               CCCGCCUUGU
Gloeophyllum trabeum            UUCGAGAUAC
Heterobasidion annosum          CUCGCGAUUC
Heterobasidion irregulare       CUCGCGAUUC
Laccaria bicolor                GCCGCAAUUC
Lentinula edodes                GUCGAAAUUU
Omphalotus olearius             GUCGCAAUUU
Phanerochaete carnosa           GCCGCCUUGU
Phanerochaete chrysosporium     GCCGCCUUGU
Piriformospora indica           GCCAAGAUUU
Pleurotus ostreatus             GUAGCCAUUU
Postia placenta                 UCCACCUUGU
Punctularia strigosozonata      GCCGUAAUUC
Rhizoctonia solani              GCCGCCAUUU
Schizophyllum commune           GCAGCCAUAG
Serpula lacrymans               UCCGCCAUUU
Stereum hirsutum                CCAGCCAUAC
Trametes versicolor             ACAGCCUUGU
"Pinus taeda"                   GCCACCUUGU
```

C

```
Tuber melanosporum
TAGTTCCTCTCCCCGATACAACCTCAAACCTCGTTTTCCCACGCTTGTACCCCTGTAGAATCC
TTCACCACGCCTTCCCCAGAGATCTACGGACCTCTGAGCCCTTCGCTTAGCGACATTACCCCA
CAACAGCCAGTCACCATTCTTCCCTCGGAACCATTGTGTTTCCCTCCAAACGACCAGCCATG

Arthrobotrys oligospora
TGAATCGCCACCCCCGCTCTCGCTCCCACCTCATCTCCAAATCCCCTTTGCCCCCCTTTTACA
AACAAAAATAGCTTCCTCTTCTTTTTTTCAAAATCAGGCCCCGTCTTTTAAAGACTCGCCTGAT
TTCACTACCGCCAAAATAACTTCGCTTAGCGAGTTACCACCCCCCCAAACCGTCAAAATTTCA
CCCTCAACGGACGCTCTGCGTTACGCACAAGCCCAAGATCCGCCAGGATG
```

Supplementary Figure 2. Conservation of NCCs in cpc-1 homologs of different fungi. (A and B) Nucleotide sequences used for generating weblogo in Figure 1 and Supplementary Figure 5, respectively. Species identifiers of the sequences used are indicated on the left. Asterisk next to species name indicates sequence presumed to derive from unknown Pezizomycotina or Agaricomycetes that is contaminating an EST library of the indicated species (e.g. Agaricomycetes sequence contaminating *Pinus taeda* library). Presumed start codons are highlighted in green. Sequences from species names in quotation marks are presumed contaminants of fungal origin because they cluster with *cpc-1* from Pezizomycotina or Agaricomycetes even though the name of the sequenced species is plant. Alignments of the 10 nucleotides including the AUG or NCC start codons and the nucleotides that define the initiation contexts (-6 to +4) which were used to generate the weblogo in Figure 1 C-J, and Supplementary Figures 5D and 5E are shown for *cpc-1/GCN4* homologs from: (A) uAUG1 uAUG2, mAUG, NCC6, NCC7 and NCC8 from Pezizomycotina; (B) the Single NCC from Agaricomycetes. (C) Nucleotide sequences of the 5' regions of *cpc-1* homologs from the upstream in-frame stop codon to the uAUG in *Tuber melanosporum* and *Arthrobotrys oligospora*. NCCs are highlighted in green.
