# Supplementary material for: Translation Initiation from Conserved Non-AUG Codons Provides Additional Layers of Regulation and Coding Capacity
Source: mBio. 2017 Jun 27;8(3):e00844-17. doi: 10.1128/mBio.00844-17 (PMC5487733; doi:10.1128/mBio.00844-17)
Supplement: FIG S3 [file mbo003173356sf3.pdf]

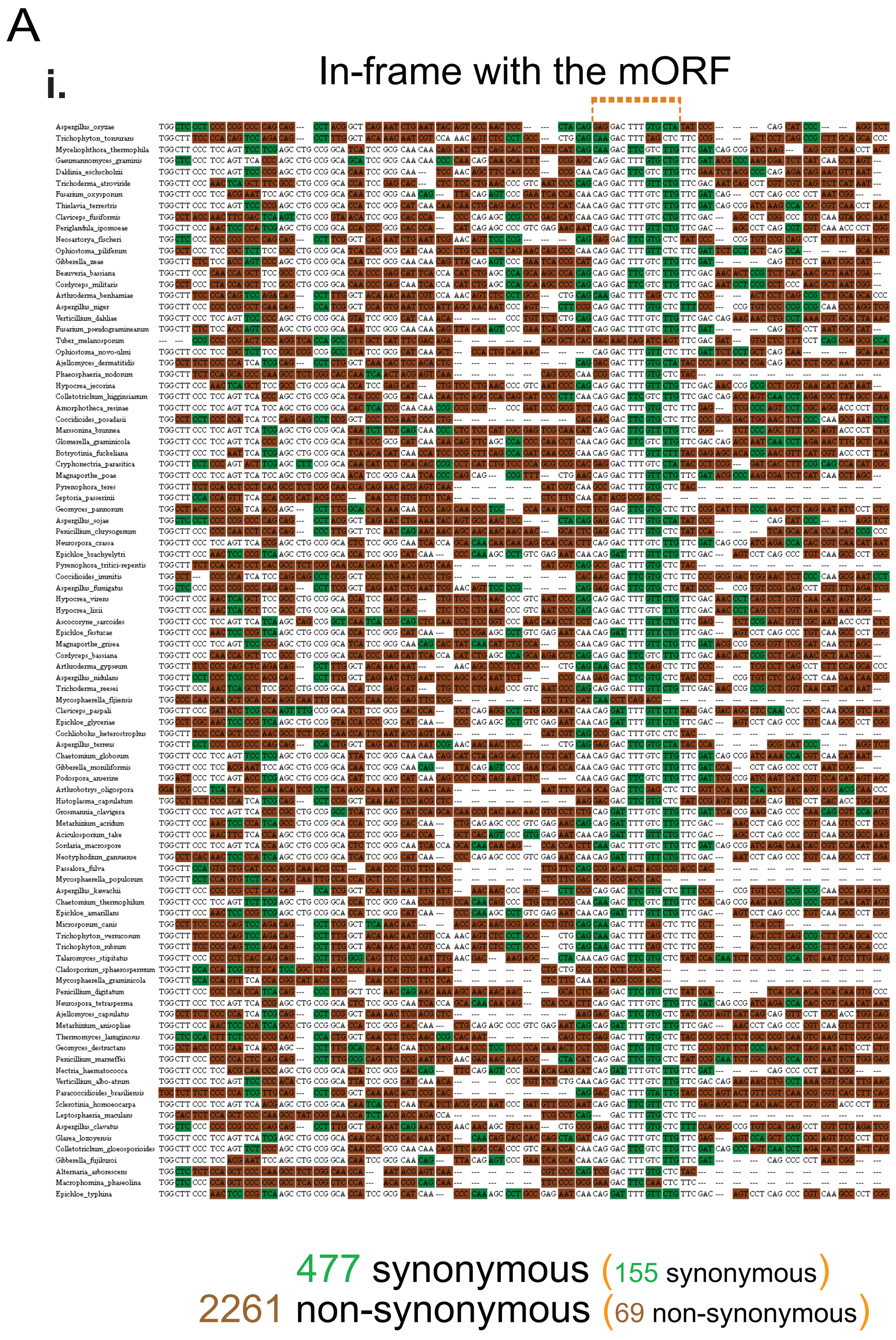
A
i.
In-frame with the mORF
477 synonymous (155 synonymous)
2261 non-synonymous (69 non-synonymous)

ii.

In-frame with uORF2

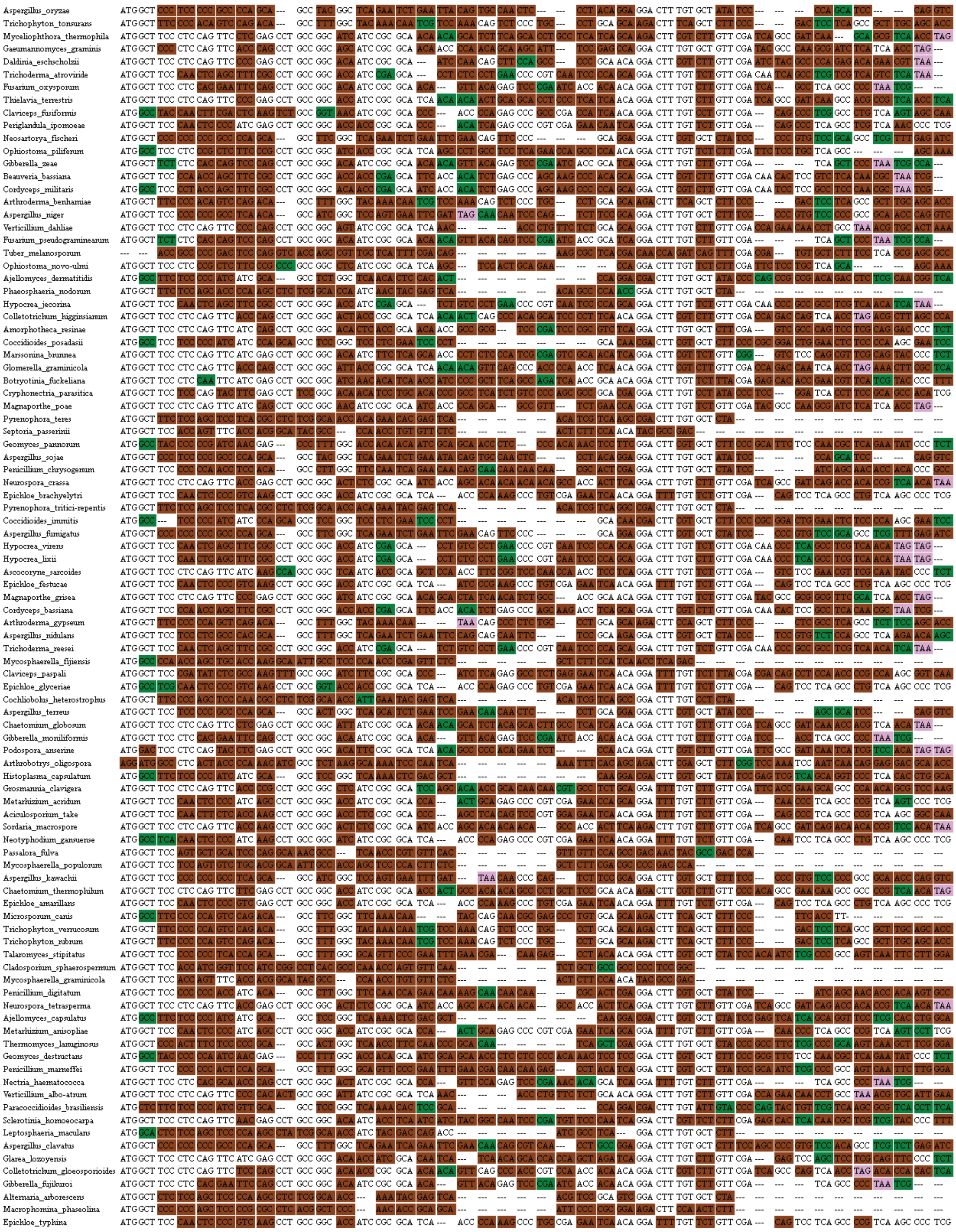

178 synonymous
2561 non-synonymous

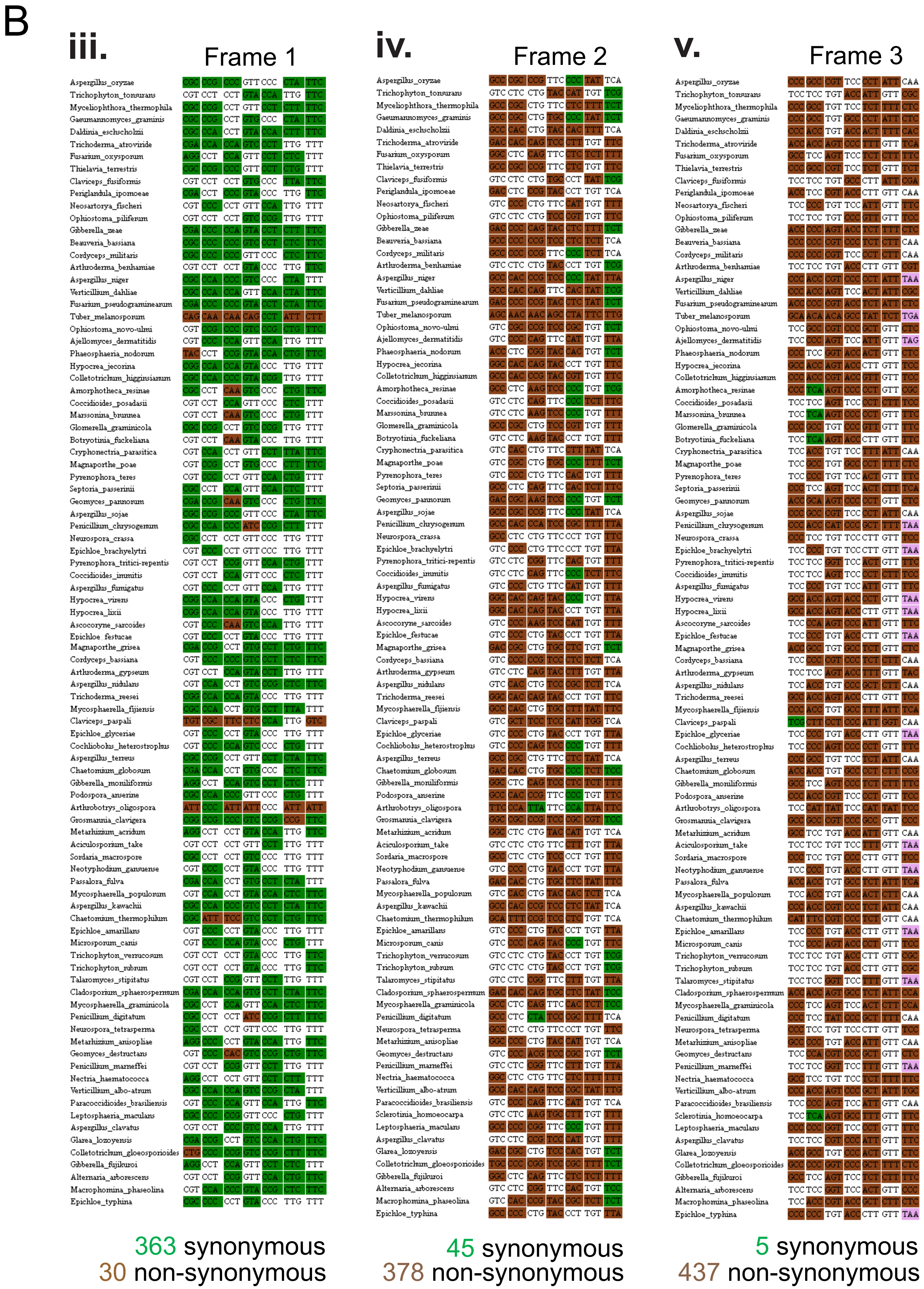
B
iii.
Frame 1
363 synonymous
30 non-synonymous
iv.
Frame 2
45 synonymous
378 non-synonymous
v.
Frame 3
5 synonymous
437 non-synonymous

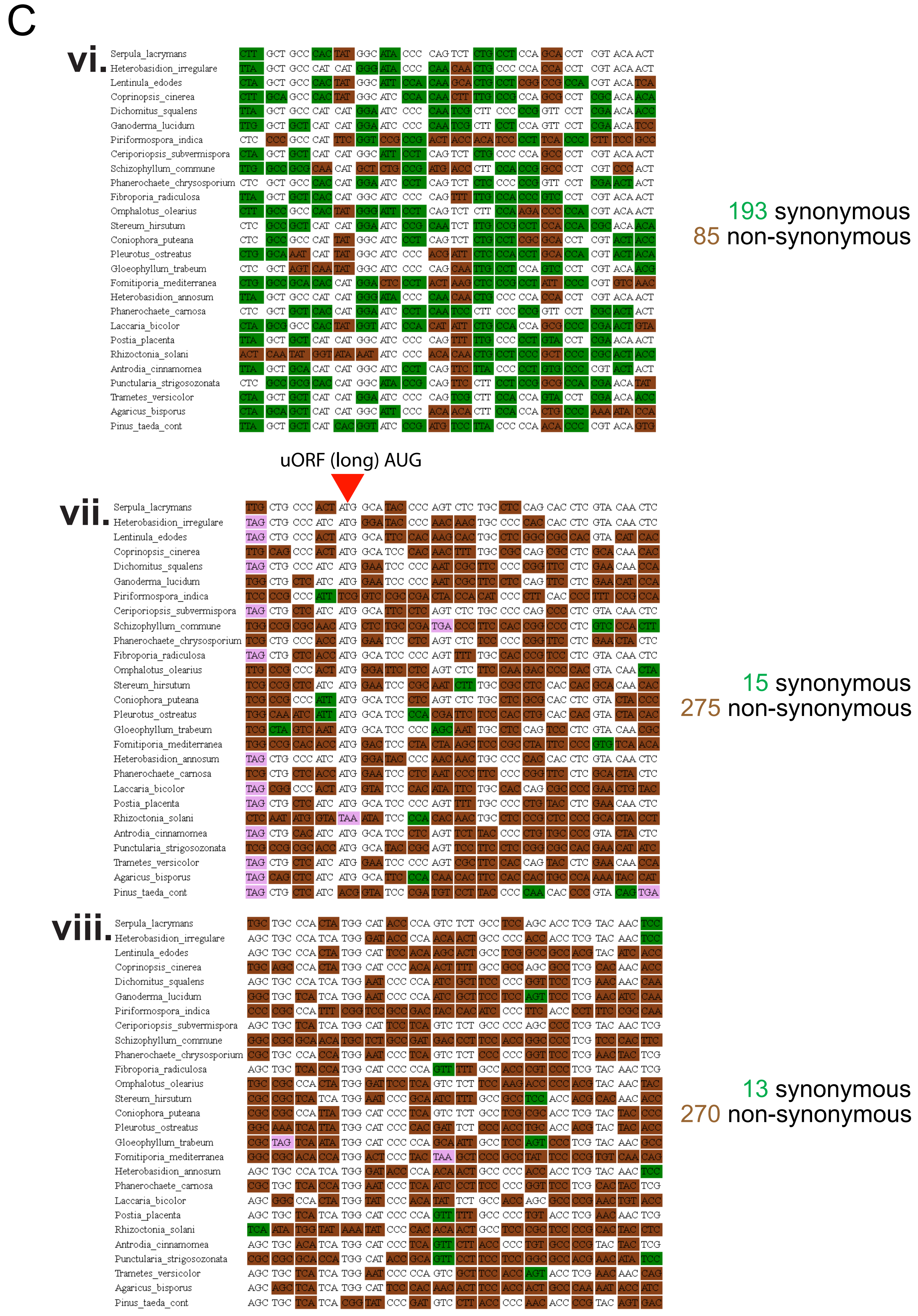
C
vi.
Serpula_lacrymans
Heterobasidion_irregulare
Lentinula_edodes
Coprinopsis_cinerea
Dichomitus_squalens
Ganoderma_lucidum
Piriformospora_indica
Ceriporiopsis_subvermispora
Schizophyllum_commune
Phanerochaete_chrysosporium
Fibroporia_radiculosa
Omphalotus_olearius
Stereum_hirsutum
Coniophora_puteana
Pleurotus_ostreatus
Gloeophyllum_trabeum
Fomitiporia_mediterranea
Heterobasidion_annosum
Phanerochaete_carnosa
Laccaria_bicolor
Postia_placenta
Rhizoctonia_solani
Antrodia_cinnamomea
Punctularia_strigosozonata
Trametes_versicolor
Agaricus_bisporus
Pinus_taeda_cont
193 synonymous
85 non-synonymous
uORF (long) AUG
vii.
15 synonymous
275 non-synonymous
viii.
13 synonymous
270 non-synonymous

Supplementary Figure 3. Regions upstream of mAUG show coding conservation in-frame with the mORF. (A) Conservation in the N-terminal extension of *N. crassa cpc-1.* Nucleotide and codon alignments of the region corresponding to uORF2 of *cpc-1* in *N. crassa* is shown in: (i) in-frame with the main ORF (frame 1 in Figure 1B); (ii) in frame with uORF2 (frame 3 in Figure 1B). (iii-v) A proline-rich region (residues 218-224, red dashed bracket in Figure 1K) upstream of the *cpc-1* mAUG shows high conservation in the mORF reading frame. Nucleotide and codon alignments of this region are shown for: (iii) frame 1 (in-frame with the mORF); (iv) frame 2; and (v) frame 3. In these alignments consensus codons (and gaps) are highlighted in white, synonymous codons in green, non-synonymous codons in brown and stop codons in magenta. Species identifiers of the sequences used are indicated on the left. The numbers of synonymous and non-synonymous substitutions are summed below each panel. The additional numbers in parentheses below panel “i” indicate the number of synonymous and non-synonymous substitutions in-frame with the mORF in the small patch of sequence marked a dashed orange bracket above the aligned sequences. This patch represents the conserved peptide region indicated by the corresponding dashed orange bracket in Figure 1K. The alignments were generated by a custom script that conceptually translated the main ORF frame into peptide sequence, then performed ClustalX alignment of peptide sequence, then converted this alignment back to nucleotides. The most common codon in each column was then designated as the consensus codon; synonymous and non-synonymous substitutions were determined relative to it. (C) The region corresponding to the beginning of uORF3 of *Coprinus cinerea cpc-1* is conserved at the amino acid level in-frame with the mORF in Agaricomycetes homologs. Nucleotide and codon alignments of the region including the beginning of uORF3 of *C. cinerea cpc-1* (indicated by red dashed lines in Supplementary Figure 5F) are shown for: (vi) frame 1 (in-frame with the main ORF); (vii) frame 2 (in-frame with uORF3); and (viii) frame 3. Species identifiers of the sequences used are indicated on the left. “Pinus_taeda_cont” indicates a sequence coming from an unknown Agaricomycetes species contaminating the *Pinus taeda* EST library. The numbers of synonymous and non-synonymous substitutions are indicated on the right of each panel. The position of the AUG start codon of uORF3 is indicated by an inverted red triangle. Coloring scheme, alignment generation and display are the same as in other panels.
