# Supplementary material for: Translation Initiation from Conserved Non-AUG Codons Provides Additional Layers of Regulation and Coding Capacity
Source: mBio. 2017 Jun 27;8(3):e00844-17. doi: 10.1128/mBio.00844-17 (PMC5487733; doi:10.1128/mBio.00844-17)
Supplement: FIG S4 [file mbo003173356sf4.pdf]

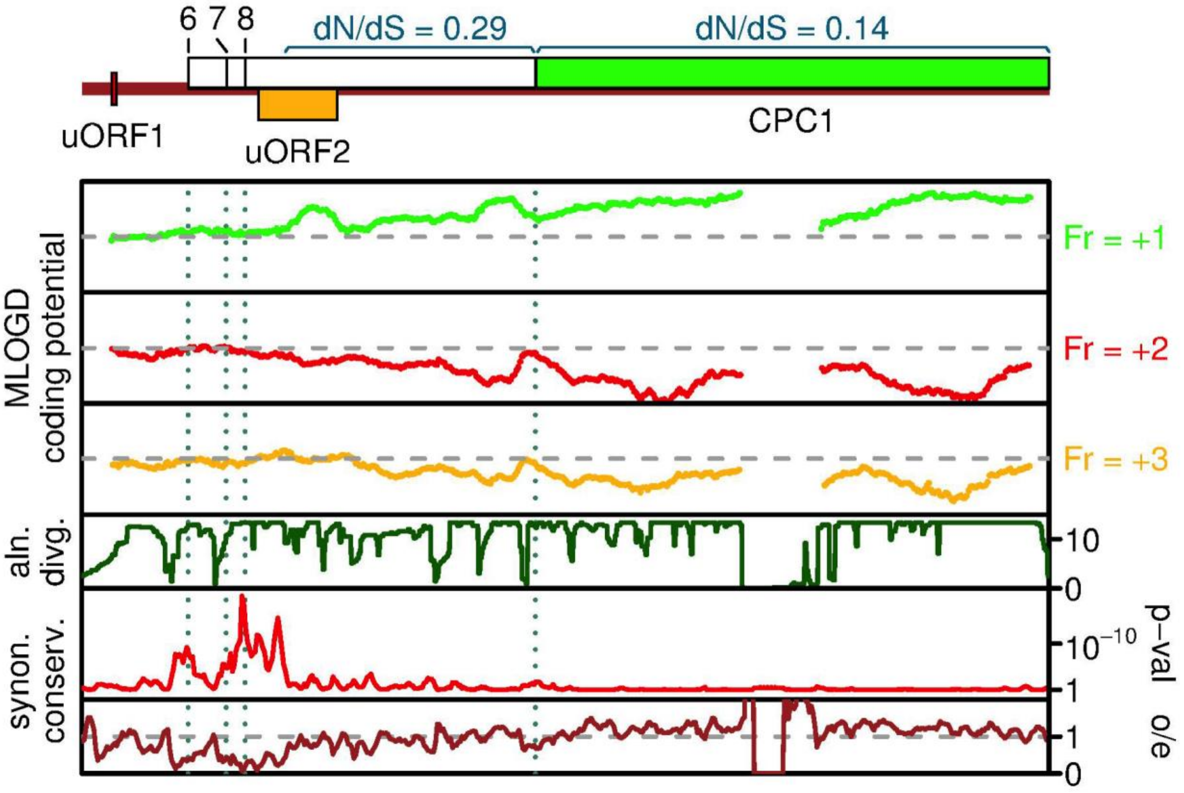

Supplementary Figure 4. Coding potential and synonymous site conservation in *cpc1*. At the top is a map of the *N. crassa* maximal CPC1-frame ORF region, showing the main CPC1 ORF, uNCCs 6, 7, and 8, and uORFs 1 and 2. Immediately below is shown the coding potential in the three reading frames as measured with MLOGD using an alignment of the maximal CPC1-frame ORF regions from 96 fungal species and a 20-codon sliding window. MLOGD uses a principle similar to the dN/dS statistic (ratio of nonsynonymous to synonymous substitutions) but also accounts for conservative amino acid substitutions being more probable than non-conservative substitutions. Positive scores indicate that the sequence is likely to be coding in the given reading frame, and are observed throughout CPC1 and most of the upstream extension, at least from uNCC-8. Positions where >50% of the alignment divergence is missing due to alignment gaps are omitted. Below this is shown alignment divergence (aln. divg., mean number of substitutions per nucleotide) for the sequences that contribute to the statistics at each position in the alignment (in any particular column, some sequences may be omitted from the statistical calculations due to alignment gaps). The bottom panels show the analysis of synonymous site conservation across the alignment. The brown line ("o/e") indicates the ratio of the observed number of synonymous substitutions within a 5-codon window to the number expected under a null model of neutral evolution at synonymous sites, while the red line ("p-val") depicts the corresponding statistical significance.
