# Supplementary material for: Translation Initiation from Conserved Non-AUG Codons Provides Additional Layers of Regulation and Coding Capacity
Source: mBio. 2017 Jun 27;8(3):e00844-17. doi: 10.1128/mBio.00844-17 (PMC5487733; doi:10.1128/mBio.00844-17)
Supplement: FIG S5 [file mbo003173356sf5.pdf]

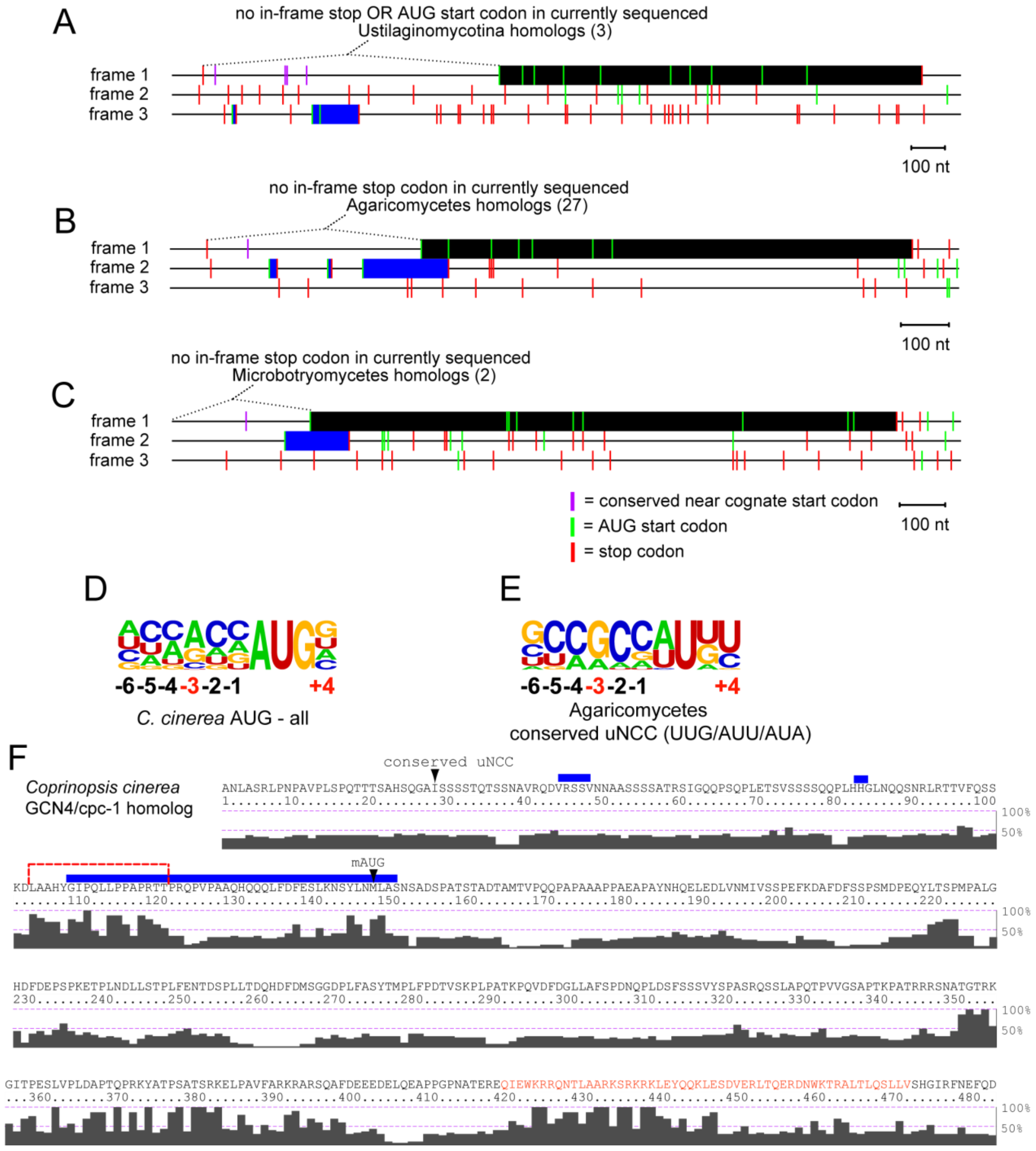

Supplementary Figure 5. Features of *cpc-1* homologs from Basidiomycota. (A-C) Diagrams of the *cpc-1* transcripts from different Basidiomycota branches. AUG codons (green bars), stop codons (red bars), mORFs (black bars) and uORFs (blue bars) are indicated. The number of sequences used for the comparisons are shown in parentheses. Features are drawn to scale. (A) *Ustilago maydis* (representing the three available Ustilaginomycotina sequences). Conserved NCCs in frame 1 upstream of uORF2 are indicated by magenta bars. (B) *Coprinopsis cinerea* (representing the 27 available Agaricomycetes sequences containing uORFs). The position of the single conserved NCC in frame 1 upstream of uORF3 is indicated by a magenta bar. (C) *Leucosporidium scottii* (representing the two available Microbotryomycetes sequences containing uORFs). The position of the single conserved NCC in frame 1 upstream of the uORF is indicated by a magenta bar. (D, E) Frequency weblogo of the conservation of the initiation contexts, from -6 to +4, of: (D) all *C. cinerea* genes; (E) the single conserved NCC in frame 1 of Agaricomycetes upstream of the uORFs. (F) The amino acid sequence encoded by frame 1 of *C. cinerea cpc-1* mRNA (the mORF frame), bounded by two in-frame stop codons. mAUG indicates the annotated initiation codon; the position of the conserved upstream NCC is also indicated. The approximate positions of uORF1, uORF2 and uORF3 are indicated by blue lines. The C-terminal b-ZIP domain is indicated in red font. The region analyzed in Supplementary Figure 3C is bracketed by a dashed red line. The conservation

of each residue from the alignment of homologs from 27 species (those used to construct the tree in Supplementary Figure 1B) is shown below the amino acid sequence.
