# Supplementary material for: Translation Initiation from Conserved Non-AUG Codons Provides Additional Layers of Regulation and Coding Capacity
Source: mBio. 2017 Jun 27;8(3):e00844-17. doi: 10.1128/mBio.00844-17 (PMC5487733; doi:10.1128/mBio.00844-17)
Supplement: FIG S6 [file mbo003173356sf6.pdf]

```
                                                               1
...AGATCGGATCCTTCCTTTCTCTTCTCTGCAACCAAATCCACCCTATCAAACCAAAACAGTAT
         BamHI                                                AC
 2        3        uORF1
CACGAGAAGCATCCATCAAGATGCGTTAAATCGCTCCCATTTCCCCGTTCTCTCGAACCCAAGTT
CACA      ACC       AAA
     4        5
CGGTAATCGTCAACATTAAACAATTTTACGTTCCCCCCTCTTCGTCAACCCCTTCAGCTCTTCCT
     ACC      TTT
            6
TCACAGGTAGATCTGAACTTCAGCACCCAGCTTCGTAGCTCGCGCTCAAGTTCTCTTACCCCCAC
      BglII CTC
       7                             8                   uORF2
CGCCACCATTCACGGCCAAGACTTTCCAGTCTTCACCACGGATTCCCAACAGTCAACATGGCTTC
       TTT                           ACA                 ACA
CCTCCAGTTCACCGAGCCTGCCGGCACTCTCCGCGCAATCACCAGCACAACAACAACAGCCACCA

CTTCAGGACTTTGTCTTGTTCGATCAGCCGATCAGACCACACCGTCAACATAACCGCAACGCTCT

CCAGCCTCCAACCCGAGGTATCAACCTGAATCAGCAACATCGTAGCCAGCATTTGTCTCCGTCTC

TGCAGAACCAGCGAGTTGCAAACATTATCCAGGCAACAGGGCACCAACTCACTTCTTCGGCTTTC

ACCAATCGGTACAGCTCTTCTCAGAACTCGCGTCCGCAACAGTTCTACGCTTCCTCAGCACCTTC

TTCAGCTTCAATCCTGAACACTCAGAACCGCGCACAGCAGCGCCCTCCTGTTCCCTTGTTTCCCA
                                        mORF
AAAGTACCGGTAGTATTTCGCACGGAAAGCAGGGCAACAAGATGTTCACTCGAGTC...
      AgeI                  TAA         CTC     XhoI
```

Supplementary Figure 6. The sequences of *cpc1-luc* fusion constructs used for in vitro experiments. Mutations and selected unique restriction sites are shown below the sequence. uORF1, uORF2 and the beginning of the main ORF (mORF) are shaded gray. NCCs are shaded magenta and are numbered 1-8. The position of the introduced TAA mutation that terminates the translation from NCCs is indicated by a red box. Luciferase was placed in-frame with CPC1 at the *Xho*I site.
