# Supplementary material for: Translation Initiation from Conserved Non-AUG Codons Provides Additional Layers of Regulation and Coding Capacity
Source: mBio. 2017 Jun 27;8(3):e00844-17. doi: 10.1128/mBio.00844-17 (PMC5487733; doi:10.1128/mBio.00844-17)
Supplement: FIG S8 [file mbo003173356sf8.pdf]

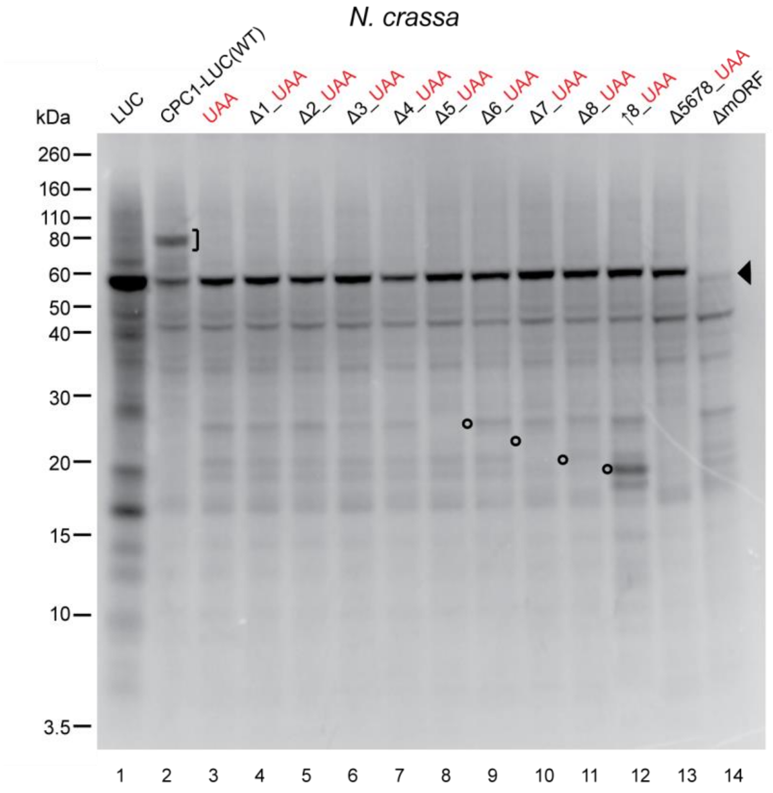

Supplementary Figure 8. Evidence from [^{35}S]Met labeling showing that NCCs 5-8, but not 1-4, initiate translation in an *N. crassa* cell-free system. Synthetic RNAs (60 ng) for the indicated constructs were used to program 10 µl of cell-free translation reactions from *N. crassa*. Reactions were incubated for 30 min at 26°C. Radiolabeled products were analyzed on 12% NuPAGE gels. Open circles: translation products eliminated upon mutation of NCCs 5-8; the product predicted to be initiated from NCC 8 also increased when NCC 8 was changed to AUG (Lane 12). Arrowhead: position of mAUG-initiated translation product (mORF). Brackets: translation products larger than the mORF produced in the absence of an in-frame UAA stop codon.
