# Supplementary material for: Translation Initiation from Conserved Non-AUG Codons Provides Additional Layers of Regulation and Coding Capacity
Source: mBio. 2017 Jun 27;8(3):e00844-17. doi: 10.1128/mBio.00844-17 (PMC5487733; doi:10.1128/mBio.00844-17)
Supplement: FIG S7 [file mbo003173356sf7.pdf]

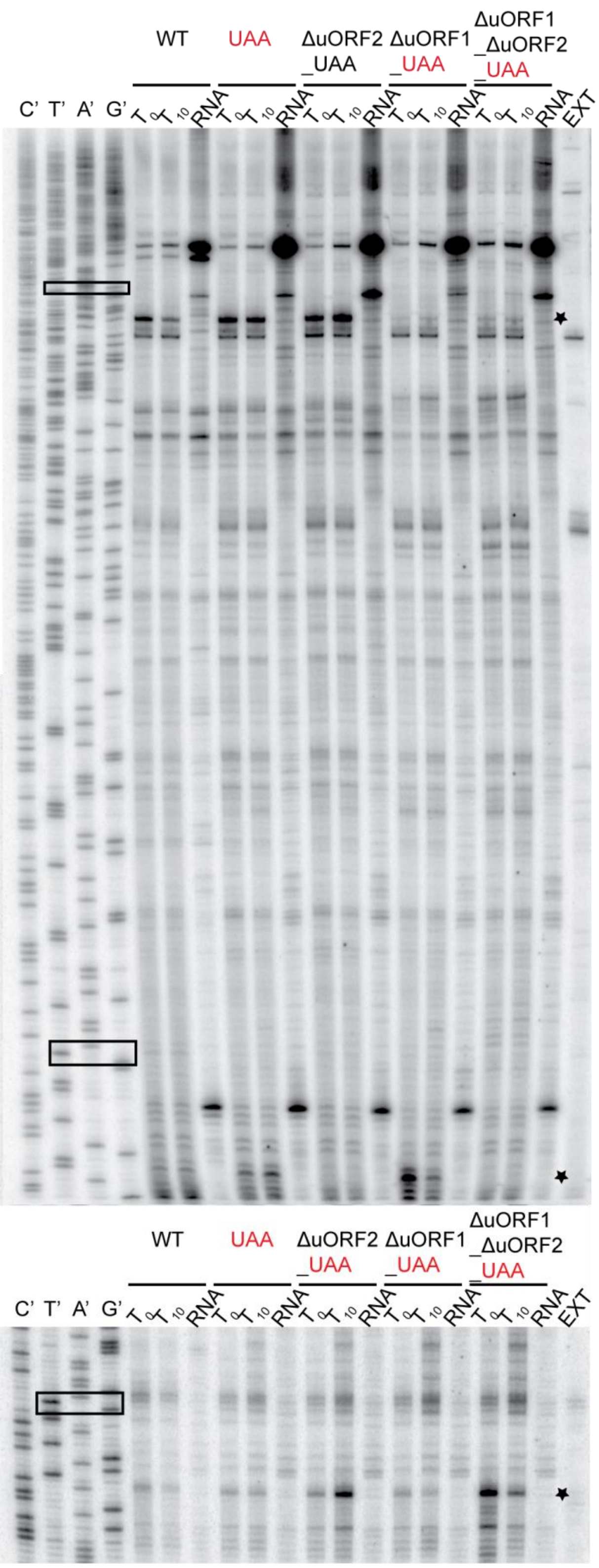

Supplementary Figure 7. Toeprint analysis indicates reinitiation following translation of *cpc-1* uORF1 but not uORF2 (primary data for Figure 2B). The description of the experiment is given in the text. For markers, either primer CPC101 (upper panel) or ZW4 (lower panel) was used to sequence the WT template (the four left-most lanes). The nucleotide complementary to the dideoxynucleotide added to each sequencing reaction for the wild-type *cpc1-luc* template is indicated above the corresponding lane so that the sequence of the template can be directly deduced; the 5’-to-3’ sequence reads from top to bottom. Asterisks (top to bottom): toeprint products corresponding to ribosomes bound at uAUG1, uAUG2 and mAUG on the *cpc1-luc* RNA. Boxes (top to bottom): uAUG1, uAUG2 and mAUG in the sequence markers. The ribosome protects approximately 16-nt 3’ of a start codon in the ribosome P-site.
