# Supplementary material for: Translation Initiation from Conserved Non-AUG Codons Provides Additional Layers of Regulation and Coding Capacity
Source: mBio. 2017 Jun 27;8(3):e00844-17. doi: 10.1128/mBio.00844-17 (PMC5487733; doi:10.1128/mBio.00844-17)
Supplement: FIG S9 [file mbo003173356sf9.pdf]

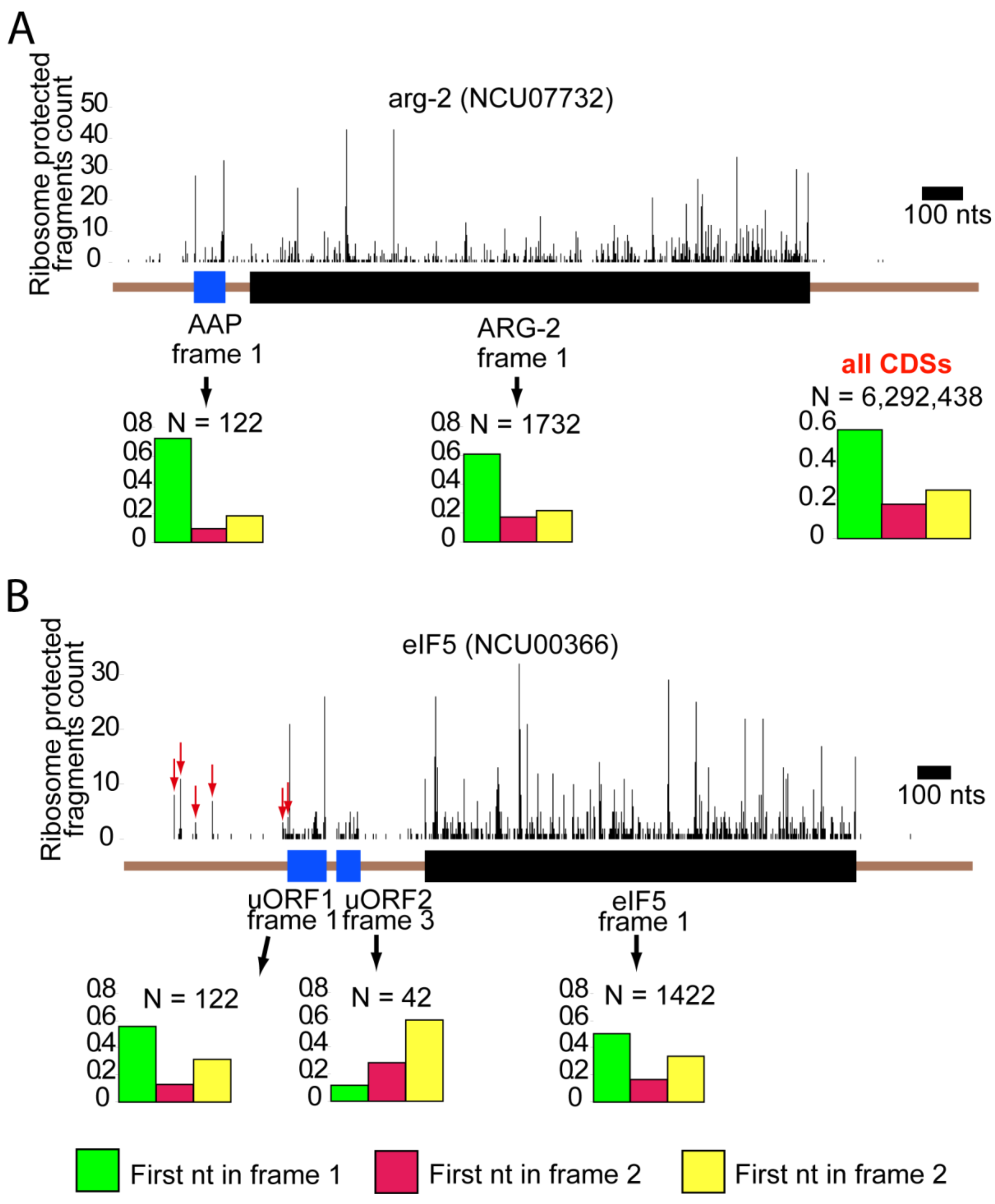

Supplementary Figure 9. Ribosome profiling data, including framing information, for *arg-2* and *eIF5*. The ribosome protected fragment count for each position 5' to 3' along the mRNA is shown on top for (A) *arg-2* (NCU07732), and (B) *eIF5* (NCU00366). Peaks corresponding to specific NCCs in the 5'-leader of *eIF5* are shown by red arrows. Schematic map of the ORF organization, drawn to scale, is shown below the ribosome protected fragment count graph. The reading frame information derived from the ribosome protected fragments for specific regions along the mRNAs is shown below the schematic maps. AAP: uORF-encoded arginine attenuator peptide; all CDSs: all predicted *N. crassa* genic ORFs. The color coding for different components of the figure are the same as in Figure 5.
