# Supplementary material for: Translation Initiation from Conserved Non-AUG Codons Provides Additional Layers of Regulation and Coding Capacity
Source: mBio. 2017 Jun 27;8(3):e00844-17. doi: 10.1128/mBio.00844-17 (PMC5487733; doi:10.1128/mBio.00844-17)
Supplement: TABLE S1 [file mbo003173356st1.pdf]

Table S1A. Plasmids used in the study.

Description	Plasmid	PCR Template	PCR primer	Cloning site	Vector
WT (CPC1-LUC)	pPC100	pPC01	BGLIIS, SACIA, SACIS, PSTIA	*Bgl*II/*Pst*I	pPC01
UAA	pPC102	pPC100	SACIS; UAA	*Sac*I/*Xho*I	pPC100
ΔuORF1_UAA	pPC187	pPC182	BamHI/S BglII/A	*Bam*HI/*Bgl*II	pPC102
ΔuORF2_UAA	pPC113	pPC100	AUG/ACA; UAA	*Sac*I/*Xho*I	pPC100
ΔuORF1_uORF2_UAA	pPC188	pPC182	BamHI/S BglII/A	*Bam*HI/*Bgl*II	pPC113
ΔuORF2	pPC104	pPC100	AUG/ACA; PSTIA	*Sac*I/*Pst*I	pPC100
ΔmAUG	pPC101	pPC100	SACIS; mAUG/ACA	*Sac*I/*Xho*I	pPC100
ΔmAUG_UAA	pPC176	AgeI/XhoI_UAA_mORF/S AgeI/XhoI_UAA_mORF/A		*Age*I/*Xho*I	pPC100
Δ8	pPC103	pPC100	ACG/ACA; PSTIA	*Sac*I/*Pst*I	pPC100
Δ5_UAA	pPC175	pPC102	Ppc1/S AUU1UUU/A AUU1UUU/S SACIA	*Bam*HI/*Bgl*II	pPC102
Δ6_UAA	pPC117	pPC100	CUG/CUC; UAA	*Bgl*II/*Xho*I	pPC100
Δ7_UAA	pPC116	pPC100	BGLIIS; AUU/UUU	*Bgl*II/*Sac*I	pPC102
Δ8_UAA	pPC114	pPC100	ACG/ACA; UAA	*Sac*I/*Xho*I	pPC100
8AUG_UAA	pPC115	pPC100	ACG/ATG; UAA	*Sac*I/*Xho*I	pPC100
ΔuORF1_Δ5_UAA	pPC179	pPC175	BamHI/S uORF1/A uORF1/S BglII/A	*Bam*HI/*Bgl*II	pPC102
ΔuORF1_Δ6_UAA	pPC180	pPC102	BamHI/S uORF1/A uORF1/S BglII/A	*Bam*HI/*Bgl*II	pPC117
ΔuORF1_Δ7_UAA	pPC181	pPC102	BamHI/S uORF1/A uORF1/S BglII/A	*Bam*HI/*Bgl*II	pPC116
ΔuORF1_Δ8_UAA	pPC182	pPC102	BamHI/S uORF1/A uORF1/S BglII/A	*Bam*HI/*Bgl*II	pPC114
ΔuORF1_8_UAA	pPC183	pPC102	BamHI/S uORF1/A uORF1/S BglII/A	*Bam*HI/*Bgl*II	pPC115
ΔuORF1_Δ5678_UAA	pPC184	pPC114	CTG/CTC_ATT2/TTT/S XhoI/A	*Bgl*II/*Xho*I	pPC179
ΔuORF1_Δ5678	pPC185	pPC103	CTG/CTC_ATT2/TTT/S XhoI/A	*Bgl*II/*Xho*I	pPC179
Δ5678	pPC186	pPC103	CTG/CTC_ATT2/TTT/S XhoI/A	*Bgl*II/*Xho*I	pPC175
In vivo WT	pJI500	pPC100		*Bam*HI/*Nsi*I	pJI401
In vivo UAA	pJI502	pPC102		*Bam*HI/*Nsi*I	pJI401
In vivo ΔmAUG	pJI501	pPC101		*Bam*HI/*Nsi*I	pJI401
In vivo ΔmAUG_UAA	pJI576	pPC176		*Bam*HI/*Nsi*I	pJI401
Vector	pJI401	pJI304 (wei,2013)	BamHI_LUC/S BstBI/A BstBI/S NsiI/A	*Bam*HI/*Nsi*I	pJI400
Vector	pJI400	Remove *BstB*I site in pJI304 by *BstB*I digestion, followed by end-repaire and self-ligation			

Table S1B. Primers used in the study.

Oligo	Sequence (5' – 3')
BGLIIS	CCCTTCAGCTCTTCCTTCACAGGT
SACIS	GCCAAGACTTTCCAGAGCTCACCACGGATTCCC
SACIA	GGGAATCCGTGGTGAGCTCTGGAAAGTCTTGGC
PSTIA	GCCTGGATAATGTTTGCAACTCGCTG
ACG/ACA	TCCAGAGCTCACCACAGATTCCCAAC
AUG/ACA	TCCAGAGCTCACCACGGATTCCCAACAGTCAACACAGCTTCCCTCC
mAUG/ACA	GTGACTCGAGTGAATGTCTTGTTGCCCT
UAA	GTGACTCGAGTGAACATCTTGTTGCCTTACTTTCCGTGCG
ACG/ATG	TCCAGAGCTCACCATGGATTCCCAAC
CUG/CUC	GGTAGATCTCAACTTCAGCACCCAG
AUU/UUU	GGTGAGCTCTGGAAAGTCTTGGCCGTGAAAGGTGGCGG
BamHI/S	TATAGATCGGATCCTTCCTTTCTCTTCTCTG
BglII/A	GAAGTTCAGATCTACCTGTGAAGGAAGAG
uORF1/S	ATCCATCAAGATGCGTTAAATCGCTCCCA
uORF1/A	TGGGAGCGATTTAACGCATCTTGATGGATGCTTC
CTG/CTC_ATT2/TTT/S	ACAGGTAGATCTGAACTTCAGCACCCAGCTTCGTAGCTCGCGCTCAAG TTCTCTTACCCCCCACCGCCACCATTCACGGCCA
XhoI/A	TCGAGTGAACATCTTGTTGCCCTGCTTTCCGTGCGAAATACTA
AgeI/XhoI_UAA_mORF/S	CCGGTAGTATTTCGCACGGAAAGTAAGGCAACAAGACATTCAC
AgeI/XhoI_UAA_mORF/A	TCGAGTGAATGTCTTGTTGCCTTACTTTCCGTGCGAAATACTA
BamHI_LUC/S	AACTAGTGGATCCTAAGCCACCAT
BstBI/S	CGAGTACTTCGAAATGTCCGTCC
BstBI/A	GGACGGACATTTCGAAGTACTCG
NsiI/A	AATGATAATGCATTGTCAGCTGTACAGTATTTAC
Ppc1/S	GGTGCATGCTAATACGACTCACTATAG
AUU1UUU/S	AATCGTCAACTTTAAACAATTTTACGTTC
AUU1UUU/A	AAATTGTTTAAAGTTGACGATTACCGAAC
SACIA	GGGAATCCGTGGTGAGCTCTGGAAAGTCTTGGC
CPC101	TCCTGAAGTGGTGGCTGTT
